# Large deletions in the *F8* gene predict immune tolerance induction failure in people with severe hemophilia A

**DOI:** 10.1016/j.rpth.2025.103212

**Published:** 2025-10-10

**Authors:** Ilja Oomen, Amal Abdi, Linda Broer, Ricardo M. Camelo, Fábia M.R.A. Callado, Luany E.M. Carvalho, Ilenia L. Calcaterra, Manuel Carcao, Giancarlo Castaman, Jeroen C.J. Eikenboom, Kathelijn Fischer, Vivian K.B. Franco, Judy Geissler, Taco W. Kuijpers, Frank W.G. Leebeek, David Lillicrap, Cláudia S. Lorenzato, Maria Elisa Mancuso, Davide Matino, Matteo N.D. Di Minno, Aomei Mo, Alex B. Mohseny, Sietse Q. Nagelkerke, Johannes Oldenburg, Suely Meireles Rezende, Georges-Etienne Rivard, Natalia Rydz, Saskia E.M. Schols, Michael W.T. Tanck, Jan Voorberg, Karin Fijnvandraat, Samantha C. Gouw, International GO-ITI Steering Group

**Affiliations:** 1 Department of Pediatric Hematology, Amsterdam UMC location University of Amsterdam, Amsterdam, the Netherlands; 2 Department of Molecular Hematology, Sanquin Research, Amsterdam, the Netherlands; 3 Department of Internal Medicine, Laboratory for Population Genomics, Human Genomics Facility, Erasmus University Medical Center, Rotterdam, the Netherlands; 4 Department of Internal Medicine, Faculty of Medicine, Universidade Federal de Minas Gerais, Belo Horizonte, Brazil; 5 Fundação de Hematologia e Hemoterapia de Pernambuco (HEMOPE), Recife, Brazil; 6 Centro de Hematologia e Hemoterapia do Ceará (HEMOCE), Fortaleza, Brazil; 7 Department of Clinical Medicine and Surgery, Federico II University, Naples, Italy; 8 Department of Pediatrics, Division of Hematology/Oncology, Hospital for Sick Children, Toronto, Canada; 9 Department of Oncology, Center for Bleeding Disorders and Coagulation, Careggi University Hospital, Florence, Italy; 10 Department of Internal Medicine, Division of Thrombosis and Hemostasis, Leiden University Medical Center, Leiden, the Netherlands; 11 Department of Hematology, Center for Benign Hematology, Thrombosis and Hemostasis, Van Creveldkliniek, University Medical Center Utrecht, Utrecht, the Netherlands; 12 Centro de Hematologia e Hemoterapia de Santa Catarina (HEMOSC), Florianópolis, Brazil; 13 Department of Blood Cell Research, Sanquin Research, Amsterdam, the Netherlands; 14 Department of Pediatric Immunology, Rheumatology and Infectious Diseases, Emma Children’s Hospital, Amsterdam UMC, University of Amsterdam, Amsterdam, Netherlands; 15 Department of Hematology, Erasmus University Medical Center, Rotterdam, the Netherlands; 16 Department of Pathology and Molecular Medicine, Queen’s University, Kingston, Ontario, Canada; 17 Coagulopathy Clinic, Hemocentro do Paraná (HEMEPAR), Curitiba, Brazil; 18 Center for Thrombosis and Hemorrhagic Diseases, IRCCS Humanitas Research Hospital, Rozzano, Milan, Italy; 19 Department of Health Research Methods, Evidence, and Impact, McMaster University, Hamilton, Ontario, Canada; 20 Department of Pediatrics, Leiden University Medical Center, Leiden, the Netherlands; 21 Institute of Experimental hematology and Transfusion Medicine, University Hospital Bonn, Medical Faculty, University of Bonn, Bonn, Germany; 22 Molecular Diagnostic Laboratory, CHU Sainte-Justine, Montréal, Québec, Canada; 23 Division of Hematology-Oncology, Department of Pediatrics, Montréal University, CHU Sainte-Justine, Montréal, Québec, Canada; 24 Division of Hematology, Department of Medicine, University of Calgary, Calgary, Canada; 25 Department of Hematology, Radboud university medical center, Nijmegen, the Netherlands; 26 Hemophilia Treatment Center Nijmegen-Eindhoven-Maastricht, Nijmegen, the Netherlands; 27 Department of Epidemiology and Data Science, Amsterdam Public Health Research Institute, Amsterdam University Medical Centers, University of Amsterdam, the Netherlands

**Keywords:** factor VIII, genetic variation, hemophilia A, immune tolerance, treatment outcome

## Abstract

**Background:**

Immune tolerance induction (ITI) is the only treatment to eradicate inhibitors in people with severe hemophilia A (SHA). Successful ITI restores factor VIII (FVIII) tolerance. ITI is demanding and successful in approximately 70% of people.

**Objectives:**

Identifying predictors of ITI outcome is essential to guide clinical decision making. We aimed to identify genetic predictors of ITI success in people with SHA and inhibitors who underwent ITI.

**Methods:**

This observational multicenter study included people with SHA who underwent ITI, between 2015 and 2023. Clinical and patient data, including FVIII gene (*F8*) mutation type, and DNA samples were collected. Successful ITI was defined by a negative inhibitor titer and an adequate response to FVIII concentrates. By employing a global screening array, the associations between ITI success and *F8* genotype and 216 candidate predictors, including single nucleotide polymorphisms and human leukocyte antigen variants, CA dinucleotide short tandem repeat polymorphisms in the *IL10* promoter region, and *FCGR2/3* gene locus variations, were analyzed.

**Results:**

Of 204 participants, 147 (72.1%) achieved ITI success. The majority (52.0%) of participants had *F8* intron 22 inversion. None of the candidate single nucleotide polymorphisms/human leukocyte antigen variants, *IL10* CA dinucleotide short tandem repeats, or *FCGR2/3* gene locus variations were associated with ITI success. *F8* large deletions were negatively associated with ITI success (odds ratio, 0.15; 95% CI, 0.04-0.51; *P* = .002).

**Conclusion:**

Our study of 204 people with SHA identified *F8* large deletions as a predictor of ITI failure. Pooling cohorts may allow the identification of additional genetic predictors of ITI success in the future.

## Essentials

• Identifying genetic predictors of immune tolerance induction success may guide shared decision making.
• In 204 people with severe hemophilia A and inhibitors, *F8* large deletions were associated with immune tolerance induction failure.

## 1 Introduction

Hemophilia A is a hereditary bleeding disorder caused by a deficiency of clotting factor VIII (FVIII). People with severe hemophilia A (SHA) (FVIII plasma activity [FVIII:C] <1 International Units [IU]/dL) suffer from spontaneous joint bleeds, resulting in joint damage, if they are not on some form of prophylaxis (FVIII or nonfactor therapies). However, neutralizing anti-FVII antibodies (referred to as inhibitors) can develop in about 25% to 35% of newly diagnosed people with SHA treated with FVIII concentrates [1]. These FVIII inhibitors hamper the procoagulant effect of administered FVIII, impeding further treatment with FVIII concentrations [2]. People with SHA traditionally develop inhibitors early in life, mostly at a median age of 15 months, after about 10 to 15 exposure days to FVIII concentrates [3,4]. The inhibitor risk decreases to <1% lifetime cumulative incidence after 50 cumulative exposure days [3].

The development of non-replacement therapies, such as emicizumab, has made prevention of most bleeds in inhibitor patients possible [5,6]. However, non-replacement therapies are not designed to treat bleeds, and bypassing agents are less effective than FVIII [7,8]. Hence, eradication of inhibitors to induce tolerance to FVIII and allow for its use to control acute bleeds is still a desirable treatment goal.

Frequent infusions of FVIII concentrates for months to years, a regimen called immune tolerance induction (ITI), is the only proven strategy to date to eradicate inhibitors. The intensity of this treatment leads to high healthcare costs [9]. Moreover, it is burdensome and demanding for both children and their parents. Nevertheless, ITI is worth trying as it has a success rate of about 70%, and inhibitor eradication leads to decreased morbidity and mortality versus the alternative of remaining inhibitor-positive [[10], [11], [12], [13]]. It is currently unknown why some patients become tolerant to FVIII, whereas others fail ITI. Therefore, there is a strong need to identify predictors of ITI success to guide clinical decision making.

Several genetic variants that are associated with inhibitor development have been reported in the literature in recent years. The large Hemophilia Inhibitor Genetics Study identified 53 genetic predictors of inhibitor development [14]. In addition, single nucleotide polymorphisms (SNPs) in genes encoding proteins involved in immune regulation and inflammation, or transcription, such as interleukin(IL)-10, tumor necrosis factor, and cytotoxic T-lymphocyte associated protein (CTLA)-4, have been reported to play a role in inhibitor development [[15], [16], [17], [18], [19]]. The rs1801274 polymorphism in the *FCGR2A* gene encoding the Fc receptor IIa has been associated with inhibitor development in people with SHA [20]. Fc gamma receptors, the immunoglobulin G receptors, may play a role in the immune response regulation by modulating the activation of antigen-presenting cells [21].

In contrast, literature on genetic predictors of ITI outcome is scarce. A recent meta-analysis reported that people with high-risk mutations (intron 22 inversion, intron 1 inversion, large deletions, and nonsense mutations) in the FVIII gene (*F8*), were less likely to achieve ITI success than those with low-risk mutations (odds ratio [OR], 0.7; 95% CI, 0.5-1.0) [22]. Previous studies reported an association between *F8* large deletions and ITI failure [23,24]. Immune checkpoint proteins, such as programmed death 1, lymphocyte activation gene 3, or CTLA-4, regulate the degree of immune activation and thereby contribute to maintenance of tolerance under normal conditions. Genetic variation in genes encoding these proteins may a play role in FVIII tolerance induction [25].

Currently, we lack significant insight into genetic determinants of ITI success that could help to identify which people with SHA and inhibitors will benefit most from ITI and who will not. Therefore, we aimed to identify genetic predictors of ITI success in people with SHA and inhibitors who underwent ITI.

## 2 Methods

### 2.1 Study design and population

This observational, retrospective, multicenter cohort study recruited children and adults with SHA (FVIII:C <1 IU/dL) who underwent primary ITI for inhibitor eradication between 2015 and 2023 in one of the participating hemophilia treatment centers (HTCs) in Brazil, Canada, the Netherlands, Germany, and Italy. All participating HTCs are listed in Supplementary Table S1. People were excluded from the current analysis if hemophilia severity was unknown, ITI was ongoing, the cumulative number of FVIII exposure days at inhibitor development exceeded 150 days, or if the DNA sample quality was poor (Figure).

**Figure:**
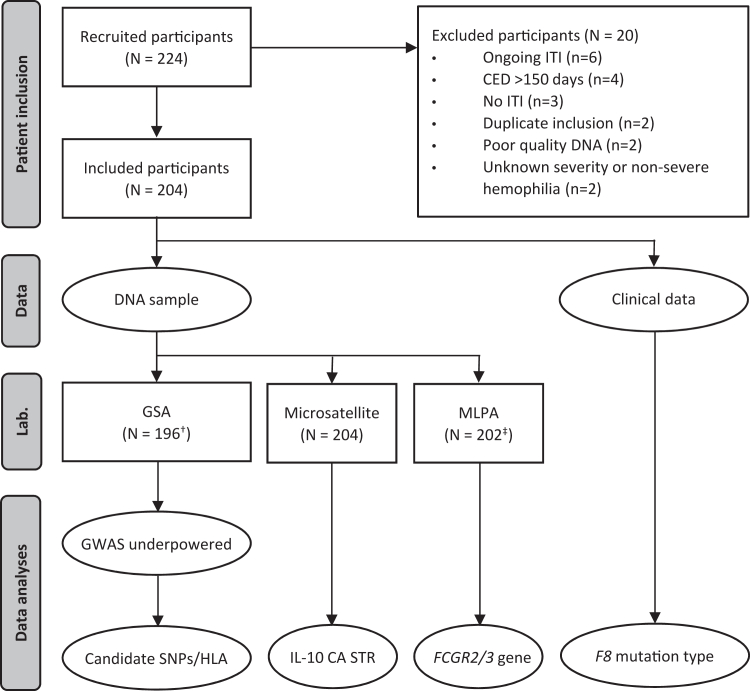
This flow chart visually presents the study methods. CED, cumulative number of factor VIII exposure days (before inhibitor development); *FCGR*, Fc gamma receptor gene; GSA, global screening array; GWAS, genome-wide association study; HLA, human leukocyte antigen; IL-10, interleukin 10; ITI, immune tolerance induction; Lab., laboratory; MLPA, multiplex ligation-dependent probe amplification; SNP, single nucleotide polymorphism; STR, short tandem repeat (CA). ^†^Eight participants were excluded due to the following reasons: no informed consent (*n* = 4), sample swab (*n* = 2), poor quality DNA sample (*n* = 1), insufficient amount of DNA (*n* = 1). ^‡^Two participants were excluded due to the following reasons: poor quality DNA sample (*n* = 1), insufficient amount of DNA (*n* = 1).

Ethical approval was obtained from ethical boards of each participating HTC. The study is registered with the number NL53406.018.15. Written informed consent was obtained from all participants or parents/guardians of minors, according to the Declaration of Helsinki.

### 2.2 Defining outcome and definitions

The study outcome was ITI success, defined by a negative inhibitor titer and clinical response to standard doses of FVIII concentrates. ITI failure was defined as failure to achieve success. Negative inhibitor titer was defined as the absence of an inhibitor titer according to the local laboratory. Clinical response to standard doses of FVIII concentrates was defined as a response that allowed bleeding to be prevented or treated. ITI regimen was defined as FVIII concentrate infusions at a dose ≥25 IU/kg at least thrice weekly. *F8* mutation type was categorized into large deletions or other mutations, as large deletion was previously associated with a high risk of inhibitor development and ITI failure [23,24,26].

### 2.3 Data collection

Clinical data were collected using study-specific case report forms. The following clinical data were collected: date of birth, baseline FVIII:C, *F8* mutation type determined in the local laboratories, ethnicity, age at inhibitor diagnosis, inhibitor titer at inhibitor diagnosis, inhibitor peak titer ever measured, inhibitor titer before ITI start, age at ITI start, ITI characteristics including type of FVIII concentrate, FVIII dose and frequency, and ITI duration.

### 2.4 Blood sampling, DNA extraction, and sample storage

Blood was drawn in 10-mL EDTA tubes and collected during a routine clinical visit to the HTC. DNA was isolated directly after blood collection in local laboratories, or EDTA blood tubes were sent to Sanquin Laboratory (Amsterdam, the Netherlands) to isolate DNA using a DNA isolation kit (Qiagen). For a subset of participants who participated in the *Hemophilia in the Netherlands* study (registration number NL59144.058.17), DNA samples were issued from the Dutch Hemophilia Biobank. DNA samples were stored at −80 °C until genetic analysis was performed.

### 2.5 Laboratory analyses

#### 2.5.1 Global screening array (GSA)

Genotyping was performed at the Human Genomics Core Facility of the Erasmus University Medical Center using the Illumina Infinium GSA-24 MD version 3.0 BeadChip. Genotyping was conducted according to the manufacturer’s instructions. Detailed information about the array is published online [27]. Genetic data were analyzed using GenomeStudio 2.0 (Illumina). Standard quality control analysis was performed using PLINK version 2.0 [28]. Imputations were performed with the 1000 Genomes Phase 3v5 reference panel [[29], [30], [31]].

#### 2.5.2 Polymorphism in *IL10* promoter region

The *IL10* CA dinucleotide short tandem repeat (STR) element was amplified by a polymerase chain reaction using 5 ng DNA and platinum SuperFi DNA polymerase (Invitrogen). Diluted polymerase chain reaction products were loaded into a 3500xl Genetic Analyzer (ThermoFisher). Data were generated using GeneMapper version 4.0 Microsatellite Analysis (ThermoFisher).

#### 2.5.3 *FCGR2/3* gene cluster

*FCGR2/*3 gene cluster genotype was assessed using multiplex ligation-dependent probe amplification as previously described [32,33]. The following functional SNPs and haplotypes were assessed: *FCGR2A* (p.His166Arg; *p.Gln62Trp*), *FCGR2B* (p.Ile232Thr; promoter haplotypes 2B.1/2B.2/2B.4), *FCGR2C* (p.Gln57Ter, as part of open reading frame/nonclassic open reading frame/Stop haplotypes; promoter haplotypes 2B.1/2B.2), *FCGR3A* (p.Val176Phe), and *FCGR3B (*haplotypes NA1/NA2/SH), as well as copy number variation in the *FCGR2C*, *FCGR3A*, and *FCGR3B* genes. Data were generated using Genemarker Version 2.6 (SoftGenetics).

### 2.6 Data analysis

Baseline characteristics were summarized by numbers and proportions, and continuous variables were reported as medians with IQRs. Pearson’s chi-squared test was used to compare categorical variables between ITI outcome groups. Fisher’s exact test was used to compare *IL10* CA dinucleotide STR between ITI outcome groups, due to small patient numbers per group. Wilcoxon rank sum test was used to compare medians with IQRs between ITI outcome groups.

Differences between the proportions of genotype/allele frequencies were compared between people with ITI success and failure using logistic regression. ORs and 95% CIs were calculated. Two-sided *P* values were calculated. For selected candidate genes, a *P* value below 2.6 × 10^−4^ (Bonferroni correction for 190 independent genetic variants, after correction for linkage disequilibrium) was considered statistically significant.

### 2.7 Genome-wide association study

A genome-wide association study (GWAS) was performed on GSA data (Supplementary Figure S3). Logistic regression was performed using Rare Variant tests to analyze the association between each SNP and ITI success. This logistic regression method was used to permit more accurate assessment of *P* values for analyzing rare variant associations [34]. In the GWAS model, we adjusted for 4 genetic principal components (PCs). In post-GWAS quality control, genetic variants with a low imputation quality (*r*^2^ < 0.3), missing information, and/or (ultra-)rare SNPs (minor allele frequency <1%) were removed. The total number of SNPs after imputation with minor allele frequency >1% and *r*^2^ > 0.3 was 10,944,149. Because this GWAS was deemed to be uninformative due to the low sample size and resulting lack of power (Supplementary Figures S3 and S4), candidate predictors were selected a priori.

### 2.8 Candidate SNPs and human leukocyte antigen variants

Candidate predictors were selected based on previously identified determinants of inhibitor development, ITI outcome, or FVIII or von Willebrand factor (VWF) pharmacokinetics in the literature. The strategy of the PubMed search for studies reporting on SNPs associated with inhibitor development is presented in Supplementary Figure S1. In addition, experts included in our Steering Group suggested a potential role for immune checkpoint-related genes to be associated with ITI outcome. Specific SNPs in these immune checkpoint-related genes reported in the literature were explored. A PubMed search was conducted to assess studies reporting on SNPs in immune checkpoint-related genes. The following search terms were used: (1) immune checkpoint-related genes, (2) polymorphism, and (3) immune therapy or autoimmunity. A total of 566 studies were screened for title and abstract, and the full text of 86 studies was screened (Supplementary Figure S2). Studies that did not find an association between the SNP and autoimmune diseases or immunotherapy outcomes were excluded. The position of each unique SNP in the genome (CGCh37) was determined by consulting the SNP database (https://www.ncbi.nlm.nih.gov/snp/). The rationale for inclusion of each variant is reported in Supplementary Tables S2 and S3. Genetic variants that were not selected as candidate predictors are reported in Supplementary Tables S4 and S5. Reasons for not including these variants were: (1) SNPs not covered by the GSA BeadChip or imputation, or (2) the specific SNP was previously reported not to be associated with inhibitor development or ITI outcome.

Linkage disequilibrium of the included SNPs was assessed using HaploReg v4.2 (Supplementary Table S2) (https://pubs.broadinstitute.org/mammals/haploreg/haploreg.php).

### 2.9 Sensitivity analysis

Principal component analysis (PCA) was used to evaluate the homogeneity of the study population. This PCA allowed selection of a genetic homogeneous population with European ancestry (Supplementary Figure S5). Genetic PCs retrieved from the PCA were used to adjust for differences in ethnic descent. Sensitivity analyses including only people with European ancestry were performed for both polymorphisms in the *IL10* promoter region and SNPs and (promoter) haplotypes in the *FCGR* gene.

### 2.10 Statistical packages

The data were prepared for analysis using SPSS Statistics version 28 (IBM). Baseline characteristics were analyzed using SPSS Statistics. Genetic data were analyzed using SPSS Statistics, RStudio version 4.2.1 (Posit), and Linux Red Hat.

## 3 Results

A total of 224 people with SHA were included (Figure). After exclusion of 20 subjects, the present study included 204 people with SHA. Reasons for exclusion were ongoing ITI (*n* = 6), cumulative number of FVIII exposure days at inhibitor developed exceeding 150 days (*n* = 4), treatment regimen not fulfilling ITI definition (*n* = 3), duplicate patient inclusion (*n* = 2), poor quality DNA sample (*n* = 2), unknown hemophilia severity (*n* = 2), and non-SHA (*n* = 1) (Figure).

Overall characteristics of the participants are shown in Table 1. Intron 22 inversion was the most prevalent *F8* mutation type (52.0%). The majority (77.0%) of participants had Caucasian ethnicity. Median age at inhibitor diagnosis and ITI start were respectively 19.8 (IQR, 12.0-38.2) and 26.1 (IQR, 15.0-61.6) months. The median inhibitor titer at inhibitor diagnosis was 5.2 (IQR, 1.5-20.0) Bethesda Units (BU)/mL. ITI was successful in 147 (72.1%) people. Distribution of characteristics was compared between people with ITI success and ITI failure. Distribution of *F8* mutation type differed between people with ITI success and ITI failure (*P* = .04). Intron 22 inversions showed comparable chances of ITI success as other *F8* mutation types, other than large deletions, which conferred the highest risk of ITI failure.

**Table 1:** Overall characteristics.

Characteristic	Total cohort (*N* = 204)	ITI outcome		
		Success (*N* = 147; 72.1%)	Failure (*N* = 57; 27.9%)	*P*
Patient characteristics				
*F8* mutation type, *n* (%)				0.037
Intron 22 inversion	106 (52.0)	77 (72.6)	29 (27.4)	
Other inversion	7 (3.4)	7 (100.0)	0 (0.0)	
Large deletion	13 (6.4)	4 (30.8)	9 (69.2)	
Nonsense mutation	27 (13.2)	20 (74.1)	7 (25.9)	
Small deletion or insertion	15 (7.4)	11 (73.3)	4 (26.7)	
Missense mutation	13 (6.4)	10 (76.9)	3 (23.1)	
Splice site mutation	2 (1.0)	2 (100.0)	0 (0.0)	
Missing, no mutation found/not determined	21 (10.3)	16 (76.2)	5 (23.8)	
Reported ethnicity, *n* (%)				
Caucasian	157 (77.0)	115 (73.2)	42 (26.8)	0.198
Hispanic/Latino	22 (10.8)	12 (54.5)	10 (45.5)	
African/African-American/African-Caribbean	8 (3.9)	7 (87.5)	1 (12.5)	
Arab/Middle Eastern	6 (2.9)	5 (83.3)	1 (16.7)	
Asian	4 (2.0)	4 (100.0)	0 (0.0)	
Other^a^	4 (2.0)	3 (75.0)	1 (25.0)	
Missing	3 (1.5)	1 (33.3)	2 (66.7)	
Inhibitor characteristics, median (IQR)				
Age at inhibitor diagnosis, mo	19.8 (12.0-38.2)	19.3 (12.2-41.7)	20.3 (11.2-33.2)	0.618
Missing, *n* (%)	18 (8.8)	12 (66.7)	6 (33.3)	
Inhibitor titer at diagnosis, BU/mL	5.2 (1.5-20.0)	4.2 (1.5-16.0)	15.0 (1.4-48.5)	0.017
Missing, *n* (%)	17 (8.3)	9 (52.9)	8 (47.1)	
Last inhibitor titer measured before ITI start (pre-ITI titer), BU/mL	6.0 (2.2-16.1)	4.5 (2.0-11.3)	14.0 (4.8-40.5)	<0.001
Missing, *n* (%)	17 (8.3)	9 (52.9)	8 (47.1)	
Inhibitor peak titer ever measured, BU/mL	51.3 (9.7-198.0)	18.2 (5.6-98.0)	180.0 (95.6-768.0)	<0.001
Missing, *n* (%)	10 (4.9)	4 (40.0)	6 (60.0)	
Inhibitor peak titer measured before ITI, BU/mL	20.4 (7.9-76.6)	13.1 (6.1-46.5)	92.9 (48.0-470.0)	<0.001
Missing, *n* (%)	92 (45.1)	59 (64.1)	33 (35.9)	
Inhibitor peak titer measured during ITI, BU/mL	82.0 (14.2-309.2)	39.5 (4.2-189.5)	172.7 (81.0-443.8)	<0.001
Missing, *n* (%)	78 (38.2)	69 (88.5)	9 (11.5)	
ITI characteristics				
Age at ITI start, mo, median (IQR)	26.1 (15.0-61.6)	26.1 (14.9-63.0)	26.0 (15.5-60.7)	0.827
Missing, *n* (%)	11 (5.4)	8 (72.7)	3 (27.3)	
FVIII product at ITI start, *n* (%)				
Recombinant FVIII	121 (59.3)	84 (69.4)	37 (30.6)	0.248
Plasma-derived FVIII	78 (38.2)	60 (76.9)	18 (23.1)	
Missing	5 (2.5)	3 (60.0)	2 (40.0)	
FVIII dose at ITI start in IU/kg/d, median (IQR)	50.0 (21.4-200.0)	59.0 (21.4-200.0)	39.4 (21.4-200.0)	0.923
Missing, *n* (%)	7 (3.4)	4 (57.1)	3 (42.9)	
ITI duration in months, median (IQR)	32.0 (19.0-52.0)	26.0 (15.0-42.0)	43.0 (33.0-73.0)	<0.001
Missing, *n* (%)	38 (18.6)	28 (73.7)	10 (26.3)	

BU, Bethesda Units; FVIII, factor VIII; ITI, immune tolerance induction; IU, International Units.

a Native-American (*n* = 2); Hispanic and Caucasian (*n* = 1); Mexican, Hispanic, and Caucasian (*n* = 1).

### 3.1 GWAS

In total, 8 (3.9%) people were excluded from either the GSA or GWAS analysis due to the following reasons: no informed consent (*n* = 4), sample swab (*n* = 2), poor quality DNA sample (*n* = 1), and insufficient amount of DNA (*n* = 1) (Figure). Characteristics of excluded people are presented in Supplementary Table S6. None of the genetic variants predicted ITI success (Supplementary Figure S3). However, our GWAS was underpowered to detect true associations between genetic variants and ITI success (Supplementary Figure S4).

### 3.2 Candidate SNPs/human leukocyte antigen variants

The selected candidate SNPs and HLA variants are presented in Supplementary Tables S2 and S3. In total, 200 candidate SNPs were selected, distributed over 80 different genes. In addition, 16 HLA variants were selected. None of the selected candidate SNPs or HLA variants were found to be significantly associated with ITI success after correction for multiple testing.

### 3.3 *IL10* CA dinucleotide STR

Logistic regression results of the *IL10* CA dinucleotide STR polymorphism in the total population are presented in Supplementary Table S7. The number of CA dinucleotide repeats was presented per allele or as sum of both alleles (STR genotype). The number of CA dinucleotide repeats was not associated with ITI success in univariate analysis or after adjustment for PCs.

### 3.4 *FCGR2/3* gene locus

Data on *FCGR2/3* gene locus variation was not available in 2 participants due to the following reasons: poor quality DNA sample (n = 1), and insufficient amount of DNA (n = 1) (Figure). Characteristics of these 2 excluded participants are presented in Supplementary Table S6.

Results of *FCGR2/3* gene locus variations on ITI success are presented in Supplementary Table S8. None of the SNPs or promoter haplotypes were associated with ITI success in univariate analysis, even after adjustment for PCs.

### 3.5 Sensitivity analysis

In sensitivity analyses, the effects of the CA dinucleotide STR polymorphism in the *IL10* promoter region and *FCGR* gene variation on ITI success were evaluated in a genetically homogenous population of people with European ancestry. In total, 113 people with SHA were considered genetically homogenous for European ancestry, based on the PCA.

Univariate analysis of *IL10* CA dinucleotide STR showed a lower chance of ITI success in people with ≥25 CA dinucleotide repeats in 1 or 2 alleles (unadjusted OR, 0.49; 95% CI, 0.24-1.00; *P* = .05) compared with people with <25 CA dinucleotide repeats (Supplementary Table S9). This weak association remained significant after adjustment for PC1-5 or PC1-3 (OR, 0.28; 95% CI, 0.10-0.72; *P* = .009 and OR, 0.37; 95% CI, 0.16-0.86; *P* = .02, respectively).

None of the SNPs and promoter haplotypes in the *FCGR* gene were associated with ITI success in the sensitivity analysis (Supplementary Table S10).

### 3.6 *F8* mutation type

People with a *F8* large deletion had a significantly lower chance of ITI success compared with those with other mutation types (OR, 0.15; 95% CI, 0.04-0.51; *P* = .002) (Table 2). Age at inhibitor diagnosis, inhibitor titer at inhibitor diagnosis, pre-ITI titer, inhibitor peak titer, and age at ITI start were higher in people with ITI failure compared with those with ITI success (Table 3). Detailed information on the type of large deletion was only available for 4 people: 2 with ITI success and 2 with ITI failure. The 2 people with large deletion and ITI success had one exon deletion (exon 1 or 14). The 2 people with large deletion and ITI failure had multiple exon deletions (exons 1-25 and exons 1-13).

**Table 2:** Effect of *F8* mutation type on ITI success, univariate analysis.

*F8* mutation type	Total cohort (*N* = 204) *n* (%)	ITI outcome		OR	95% CI	*P*
		Success (*N* = 147) *n* (%)	Failure (*N* = 57) *n* (%)			
Other mutation type	170 (83.3)	127 (74.7)	43 (25.3)	*ref*	*ref*	
Large deletion	13 (6.4)	4 (30.8)	9 (69.2)	0.15	0.04-0.51	0.002
Missing	21 (10.3)	16 (76.2)	5 (23.8)			

OR = odds ratio, ref = reference.

**Table 3:** Characteristics of persons with *F8* large deletions, categorized per ITI outcome.

Characteristic	Success (*N* = 4) *n* (%)	Failure (*N* = 9) *n* (%)
Age at inhibitor diagnosis, mo, median (IQR)	31.2 (16.1-99.8)	37.7 (19.4-50.7)
Inhibitor titer at inhibitor diagnosis, BU/mL, median (IQR)	8.6 (0.4-244.0)	14.0 (5.1-45.6)
Last inhibitor titer measured before ITI start (pre-ITI titer), BU/mL, median (IQR)	13.3 (3.6-97.0)	34.0 (14.7-135.9)
Inhibitor peak titer ever measured, BU/mL, median (IQR)	25.0 (9.5-623.8)	819.0 (119.1-2688.0)
Age at ITI start, mo, median (IQR)	45.1 (17.5-142.2)	88.1 (23.1-282.8)

BU, Bethesda Units; ITI, immune tolerance induction.

## 4 Discussion

This international multicenter study of 204 people with SHA and inhibitors who underwent ITI assessed potential genetic variants of ITI success. The effects of 216 candidate SNPs/HLA variants, CA dinucleotide STR in the *IL10* promoter region, *FCGR2/3* gene locus variations and *F8* mutation type on ITI success were assessed. *F8* large deletions were associated with ITI failure in an univariate analysis.

In contrast to the scarce knowledge on potential genetic predictors of ITI outcome, genetic predispositions for inhibitor development have been extensively investigated. The Hemophilia Inhibitor Genetics Study investigated the association between 13,331 SNPs, primarily located in immune regulatory genes, and inhibitor development [14]. This study identified 53 significant predictors for inhibitor development. Furthermore, Astermark et al. [15] identified an association between 20 CA dinucleotide STR in the promoter region of *IL10* and inhibitor formation (134 bp = 20 CA repeats). We found a potential association between ≥25 CA dinucleotide STR and ITI failure in a genetically homogenous population of people with European ancestry (unadjusted OR, 0.49; 95% CI, 0.24-1.00; *P* = .05). Whether this polymorphism affects IL-10 secretion *in vivo*, directing the immune response, remains unclear. Additionally, SNPs in genes encoding pro- or anti-inflammatory enzymes or cytokines, such as heme oxygenase 1 (*HMOX1*), tumor necrosis factor-ɑ, IL-10, and CTLA-4, were previously associated with inhibitor development [[15], [16], [17],^35^]. A potential role for the Fc gamma receptor on inhibitor development was also previously investigated, identifying an association between *FCGR2A* p.166His and inhibitor development [20]. In our study, we did not find an association between *FCGR* gene variations and ITI outcome, potentially due to the small sample size or because the genetic variations in these genes may not contribute substantially toward ITI success or failure. Another study demonstrated that patients who developed inhibitors were less likely to express indoleamine 2,3-dioxygenase 1, encoded by the *IDO1* gene, a key regulatory enzyme involved in regulatory T cell functions and peripheral tolerance [36]. More recently, B-cell activating factor was found to modulate FVIII inhibitor formation in humans and mice with hemophilia A [37]. None of the candidate SNPs in the abovementioned genes were associated with ITI outcome in our study.

The relationship between the type of *F8* mutation and the risk of inhibitor development has been a major focus of research. A meta-analysis by Gouw et al. [26] reported the highest risk of inhibitor development in people with large deletions (pooled OR, 3.6; 95% CI, 2.3-5.7), in line with the results of the PedNet cohort among 1202 children with SHA [38]. Recently, the association between *F8* mutation type and ITI outcome has been investigated [23,24]. *F8* large deletions have been reported to confer the highest risk of ITI failure [23]. Consistent with these results, our study demonstrated a 0.15-fold (95% CI, 0.04-0.51; *P* = .002) reduced chance of ITI success in people with *F8* large deletions compared with those with other *F8* mutation types, translating to a 6.6-fold increased risk of ITI failure (95% CI, 1.95-22.68). Intracellular production of portions of the endogenous FVIII protein may lead to some degree of tolerance to exogenous FVIII proteins, which reduces the risk of inhibitor development [26]. Indeed, large deletions causing complete absence of FVIII confer the highest risk of inhibitor development [26]. Perhaps, exposure to residual endogenous FVIII by antigen-presenting cells can also explain the higher chance of ITI success in people with other *F8* mutation types than large deletions, as observed for intron 22 inversions in our study.

The observations of the Cohorts for Heart and Aging Research in Genomic Epidemiology (CHARGE) Consortium has yielded unexpected insights into processes involved in the clearance and immunogenicity of FVIII [39,40]. The C-type lectin CLEC4M receptor plays a role in regulating plasma levels of FVIII and VWF [41,42]. Another clearance receptor identified by the CHARGE consortium was stabilin-2, encoded by the *STAB2* gene [43]. The effects of SNPs in both receptors, in addition to genes involved in regulation of FVIII or VWF plasma levels, on ITI outcome were investigated in the present study; however, none of the candidate predictors were found to be associated with ITI outcome.

To our knowledge, this is the first study to examine over 200 genetic candidate predictors of ITI success in a large cohort of people with SHA who underwent ITI. We have attempted to use a hypothesis-free approach by employing the GSA to identify possible new candidate genes of ITI success. Nevertheless, due to the restricted number of included participants, we were obligated to select candidate predictors on the basis of available literature. The My Life, Our Future cohort identified large deletions and complex intron 22 inversions associated with the highest inhibitor rate [44]. Unfortunately, we did not have additional information on the *F8* intron 22 inversion subtype. For further studies, we will specify on the case report form to obtain the most comprehensive genetic data from participating centers. We aimed to select genetic variants that were previously associated with inhibitor development or FVIII or VWF pharmacokinetics, but it is possible that these polymorphisms do not play a role in the underlying mechanism of ITI. However, because ITI is not comparable to any other immunotherapy or immune-mediated diseases, this was the best possible rationale for SNP selection. Nevertheless, our study remained underpowered to detect true associations of ITI success. In this regard, we chose not to adjust for ethnicity in the GWAS analysis of GSA data. Therefore, it is possible that we may have missed potential associations of ITI success due to ethnical heterogeneity. The number of reported people with Caucasian ethnicity was higher than the number of individuals genetically identified as having European ancestry. This may be explained by the fact that skin color, rather than ethnicity, was reported in people recruited from the HTC in Brazil. Therefore, white skin color was classified as Caucasian ethnicity, but the Caucasian classification likely does not reflect Brazilian whites [45].

Inhibitor development leads to increased morbidity and mortality and remains the most severe complication of FVIII treatment in people with SHA [11,46,47]. Therefore, inhibitor eradication remains a treatment goal for all people with SHA and inhibitors, unless the chances of ITI success are very low. Identification of genetic predictors of ITI success will allow for better assignment of people with high or low probabilities of ITI success. We identified *F8* large deletions (multiple exons) as a predictor for ITI failure. ITI success is determined by an interplay between genetic and nongenetic risk factors. In addition to clinical predictors, *F8* large deletions could contribute to a prediction score to estimate the chance of ITI success. In people with *F8* large deletions covering multiple exons, who also have other poor prognostic factors of ITI success such as very high peak inhibitor titers (>200 BU/mL), ITI should perhaps not be attempted, as the chances of ITI success are very low. Although representing a large cohort, our study was underpowered to reliably identify genetic predictors of ITI success. However, this study is the first step in exploring genetic determinants of ITI success. In the future, our data may be pooled with data from other cohorts, which could increase the power to hopefully allow identification of genetic determinants of ITI success.

## References

[1] Wight J., Paisley S. (2003). The epidemiology of inhibitors in haemophilia A: a systematic review. Haemophilia.

[2] Ananyeva N.M., Lacroix-Desmazes S., Hauser C.A.E., Shima M., Ovanesov M.V., Khrenov A.V. (2004). Inhibitors in hemophilia A: mechanisms of inhibition, management and perspectives. Blood Coagul Fibrinolysis.

[3] Gouw S.C., van der Bom J.G., van den Berg H.M. (2007). Treatment-related risk factors of inhibitor development in previously untreated patients with hemophilia A: the CANAL cohort study. Blood.

[4] Gouw S.C., van der Bom J.G., Ljung R., Escuriola C., Cid A.R., Claeyssens-Donadel S. (2013). Factor VIII products and inhibitor development in severe hemophilia A. N Engl J Med.

[5] Oldenburg J., Mahlangu J.N., Kim B., Schmitt C., Callaghan M.U., Young G. (2017). Emicizumab prophylaxis in hemophilia A with inhibitors. N Engl J Med.

[6] Mahlangu J.N., Oldenburg J., Paz-Priel I., Negrier C., Niggli M., Mancuso M.E. (2018). Emicizumab prophylaxis in patients who have hemophilia A without inhibitors. N Engl J Med.

[7] Witmer C., Young G. (2013). Factor VIII inhibitors in hemophilia A: rationale and latest evidence. Ther Adv Hematol.

[8] Young G. (2018). Implementing emicizumab in hemophilia inhibitor management: emicizumab should be prescribed after tolerance. Blood Adv.

[9] Gringeri A., Mantovani L.G., Scalone L., Mannucci P.M., COCIS Study Group (2003). Cost of care and quality of life for patients with hemophilia complicated by inhibitors: the COCIS Study Group. Blood.

[10] Eckhardt C.L., Loomans J.I., van Velzen A.S., Peters M., Mauser-Bunschoten E.P., Schwaab R. (2015). Inhibitor development and mortality in non-severe hemophilia A. J Thromb Haemost.

[11] Walsh C.E., Soucie J.M., Miller C.H., United States Hemophilia Treatment Center Network (2015). Impact of inhibitors on hemophilia A mortality in the United States. Am J Hematol.

[12] Shapiro A.D., Mitchell I.S., Nasr S. (2018). The future of bypassing agents for hemophilia with inhibitors in the era of novel agents. J Thromb Haemost.

[13] Santagostino E., Young G., Escuriola Ettingshausen C., Jiménez-Yuste V., Carcao M. (2019). Inhibitors: a need for eradication?. Acta Haematol.

[14] Astermark J., Donfield S.M., Gomperts E.D., Schwarz J., Menius E.D., Pavlova A. (2013). The polygenic nature of inhibitors in hemophilia A: results from the Hemophilia Inhibitor Genetics Study (HIGS) Combined Cohort. Blood.

[15] Astermark J., Oldenburg J., Pavlova A., Berntorp E., Lefvert A.K., MIBS Study Group (2006). Polymorphisms in the *IL10* but not in the *IL1β* and *IL4* genes are associated with inhibitor development in patients with hemophilia A. Blood.

[16] Astermark J., Oldenburg J., Carlson J., Pavlova A., Kavakli K., Berntorp E. (2006). Polymorphisms in the *TNFA* gene and the risk of inhibitor development in patients with hemophilia A. Blood.

[17] Astermark J., Wang X., Oldenburg J., Berntorp E., Lefvert A.K., MIBS Study Group (2007). Polymorphisms in the CTLA-4 gene and inhibitor development in patients with severe hemophilia A. J Thromb Haemost.

[18] Astermark J., Berntorp E., White G.C., Kroner B.L., MIBS Study Group (2001). The Malmö International Brother Study (MIBS): further support for genetic predisposition to inhibitor development in hemophilia patients. Haemophilia.

[19] Bachelet D., Albert T., Mbogning C., Hässler S., Zhang Y., Schultze-Strasser S. (2019). Risk stratification integrating genetic data for factor VIII inhibitor development in patients with severe hemophilia A. PLoS One.

[20] Eckhardt C.L., Astermark J., Nagelkerke S.Q., Geissler J., Tanck M.W., Peters M. (2014). The Fc gamma receptor IIa R131H polymorphism is associated with inhibitor development in severe hemophilia A. J Thromb Haemost.

[21] Nimmerjahn F., Ravetch J.V. (2008). Fcγ receptors as regulators of immune responses. Nat Rev Immunol.

[22] Oomen I., Camelo R.M., Rezende S.M., Voorberg J., Mancuso M.E., Oldenburg J. (2023). Determinants of successful immune tolerance induction in hemophilia A: systematic review and meta-analysis. Res Pract Thromb Haemost.

[23] Zuccherato L.W., Souza R.P., Camelo R.M., Dias M.M., Jardim L.L., Santana M.A.P. (2024). Large deletions and small insertions and deletions in the factor VIII gene predict unfavorable immune tolerance induction outcome in people with severe hemophilia A and high-responding inhibitors. Thromb Res.

[24] Coppola A., Margaglione M., Santagostino E., Rocino A., Grandone E., Mannucci P.M. (2009). Factor VIII gene (*F8*) mutations as predictors of outcome in immune tolerance induction of hemophilia A patients with high-responding inhibitors. J Thromb Haemost.

[25] Wagner M., Jasek M., Karabon L. (2020). Immune checkpoint molecules—inherited variations as markers for cancer risk. Front Immunol.

[26] Gouw S.C., van den Berg H.M., Oldenburg J., Astermark J., de Groot P.G., Margaglione M. (2012). *F8* gene mutation type and inhibitor development in patients with severe hemophilia A: systematic review and meta-analysis. Blood.

[27] Illumina (2020).

[28] Purcell S., Neale B., Todd-Brown K., Thomas L., Ferreira M.A.R., Bender D. (2007). PLINK: a tool set for whole-genome association and population-based linkage analyses. Am J Hum Genet.

[29] Das S., Forer L., Schönherr S., Sidore C., Locke A., Kwong A. (2016). Next-generation genotype imputation service and methods. Nat Genet.

[30] Loh P.R., Danecek P., Palamara P.F., Fuchsberger C., Reshef Y.A., Finucane H.K. (2016). Reference-based phasing using the Haplotype Reference Consortium panel. Nat Genet.

[31] Auton A., Brooks L.D., Durbin R.M., Garrison E.P., Kang H.M., 1000 Genomes Project Consortium (2015). A global reference for human genetic variation. Nature.

[32] Nagelkerke S.Q., Tacke C.E., Breunis W.B., Tanck M.W.T., Geissler J., Png E. (2019). Extensive ethnic variation and linkage disequilibrium at the *FCGR2/3* locus: different genetic associations revealed in Kawasaki disease. Front Immunol.

[33] Schouten J.P., McElgunn C.J., Waaijer R., Zwijnenburg D., Diepvens F., Pals G. (2002). Relative quantification of 40 nucleic acid sequences by multiplex ligation-dependent probe amplification. Nucleic Acids Res.

[34] Zhan X., Hu Y., Li B., Abecasis G.R., Liu D.J. (2016). RVTESTS: an efficient and comprehensive tool for rare variant association analysis using sequence data. Bioinformatics.

[35] Repessé Y., Peyron I., Dimitrov J.D., Dasgupta S., Moshai E.F., Costa C. (2013). Development of inhibitory antibodies to therapeutic factor VIII in severe hemophilia A is associated with microsatellite polymorphisms in the HMOX1 promoter. Haematologica.

[36] Matino D., Gargaro M., Santagostino E., Di Minno M.N.D., Castaman G., Morfini M. (2015). IDO1 suppresses inhibitor development in hemophilia A treated with factor VIII. J Clin Invest.

[37] Doshi B.S., Rana J., Castaman G., Shaheen M.A., Kaczmarek R., Butterfield J.S.S. (2021). B cell–activating factor modulates the factor VIII immune response in hemophilia A. J Clin Invest.

[38] Andersson N.G., Labarque V., Kartal-Kaess M., Pinto F., Mikkelsen T.S., Ljung R. (2024). Factor VIII genotype and the risk of developing high-responding or low-responding inhibitors in severe hemophilia A: data from the PedNet Hemophilia Cohort of 1,202 children. Haematologica.

[39] Smith N.L., Chen M.H., Dehghan A., Strachan D.P., Basu S., Soranzo N. (2010). Novel associations of multiple genetic loci with plasma levels of factor VII, factor VIII, and von Willebrand factor: The CHARGE (Cohorts for Heart and Aging Research in Genome Epidemiology) Consortium. Circulation.

[40] Lacroix-Desmazes S., Voorberg J., Lillicrap D., Scott D.W., Pratt K.P. (2020). Tolerating factor VIII: recent progress. Front Immunol.

[41] Swystun L.L., Notley C., Georgescu I., Lai J.D., Nesbitt K., James P.D. (2019). The endothelial lectin clearance receptor CLEC4M binds and internalizes factor VIII in a VWF-dependent and independent manner. J Thromb Haemost.

[42] Rydz N., Swystun L.L., Notley C., Paterson A.D., Riches J.J., Sponagle K. (2013). The C-type lectin receptor CLEC4M binds, internalizes, and clears von Willebrand factor and contributes to the variation in plasma von Willebrand factor levels. Blood.

[43] Swystun L.L., Lai J.D., Notley C., Georgescu I., Paine A.S., Mewburn J. (2018). The endothelial cell receptor stabilin-2 regulates VWF-FVIII complex half-life and immunogenicity. J Clin Invest.

[44] Johnsen J.M., Fletcher S.N., Dove A., McCracken H., Martin B.K., Kircher M. (2022). Results of genetic analysis of 11 341 participants enrolled in the My Life, Our Future hemophilia genotyping initiative in the United States. J Thromb Haemost.

[45] Queiroz E.M., Santos A.M., Castro I.M., Machado-Coelho G.L.L., Cândido A.P.C., Leite T.M. (2013). Genetic composition of a Brazilian population: the footprint of the Gold Cycle. Genet Mol Res.

[46] Morfini M., Haya S., Tagariello G., Pollmann H., Quintana M., Siegmund B. (2007). European study on orthopaedic status of haemophilia patients with inhibitors. Haemophilia.

[47] Monahan P.E., Baker J.R., Riske B., Soucie J.M. (2011). Physical functioning in boys with hemophilia in the U.S. Am J Prev Med.

