# Supplementary material for: Large deletions in the F8 gene predict immune tolerance induction failure in people with severe hemophilia A
Source: Res Pract Thromb Haemost. 2025 Oct 10;9(7):103212. doi: 10.1016/j.rpth.2025.103212 (PMC12616064; doi:10.1016/j.rpth.2025.103212)
Supplement: Supplementary material [file mmc1.pdf]

Supplementary material of "Large deletions in the *F8* gene predict immune tolerance induction failure in people with severe hemophilia A"

Authors

Ilja Oomen[1,2], Amal Abdi[1], Linda Broer[3], Ricardo M. Camelo[4], Fábia M.R.A. Callado[5], Luany E.M. Carvalho[6], Ilenia L. Calcaterra[7], Manuel Carcao[8], Giancarlo Castaman[9], Jeroen C.J. Eikenboom[10], Kathelijn Fischer[11], Vivian K.B. Franco[12], Judy Geissler[13], Taco W. Kuijpers[13,14], Frank W.G. Leebeek[15], David Lillicrap[16], Cláudia S. Lorenzato[17], Maria Elisa Mancuso[18], Davide Matino[19], Matteo N.D. Di Minno[7], Aomei Mo[16], Alex B Mohseny[20], Sietse Q. Nagelkerke[13,14], Johannes Oldenburg[21], Suely Meireles Rezende[4], Georges-Etienne Rivard[22,23], Natalia Rydz[24], Saskia E.M. Schols[25,26], Michael M.T. Tanck[27], Jan Voorberg[2], Karin Fijnvandraat[1,2], Samantha C Gouw[1], on behalf of the International GO-ITI Steering Group.

Affliations

1. Amsterdam UMC location University of Amsterdam, Department of Pediatric Hematology, Meibergdreef 9, Amsterdam, the Netherlands
2. Department of Molecular Hematology, Sanquin Research, Amsterdam, the Netherlands
3. Department of Internal Medicine, Laboratory for Population Genomics, Human Genomics Facility, Erasmus University Medical Center, Rotterdam, the Netherlands
4. Department of Internal Medicine, Faculty of Medicine, Universidade Federal de Minas Gerais, Belo Horizonte, Brazil
5. Fundação de Hematologia e Hemoterapia de Pernambuco (HEMOPE), Recife, Brazil
6. Centro de Hematologia e Hemoterapia do Ceará (HEMOCE), Fortaleza, Brazil
7. Department of Clinical Medicine and Surgery, Federico II University, Naples, Italy
8. Department of Pediatrics, Division of Hematology/Oncology, Hospital for Sick Children, Toronto, Canada
9. Department of Oncology, Center for Bleeding Disorders and Coagulation, Careggi University Hospital, Florence, Italy
10. Department of Internal Medicine, Division of Thrombosis and Hemostasis, Leiden University Medical Center, Leiden, the Netherlands
11. Department of Hematology, Center for Benign Hematology, Thrombosis and Hemostasis, Van Creveldkliniek, University Medical Center Utrecht, Utrecht, the Netherlands
12. Centro de Hematologia e Hemoterapia de Santa Catarina (HEMOSC), Florianópolis, Brazil
13. Department of Blood Cell Research, Sanquin Research, Amsterdam, the Netherlands

14. Department of Pediatric Immunology, Rheumatology and Infectious Diseases, Emma Children's Hospital, Amsterdam UMC, University of Amsterdam, Amsterdam, Netherlands
15. Department of Hematology, Erasmus University Medical Center, Rotterdam, the Netherlands
16. Department of Pathology and Molecular Medicine, Queen's University, Kingston, Ontario, Canada
17. Coagulopathy Clinic, Hemocentro do Paraná (HEMEPAR), Curitiba, Brazil
18. Center for Thrombosis and Hemorrhagic Diseases, IRCCS Humanitas Research Hospital, Rozzano, Milan, Italy
19. Department of Health Research Methods, Evidence, and Impact (HEI), McMaster University, Hamilton, Ontario, Canada
20. Department of Pediatrics, Leiden University Medical Center, Leiden, the Netherlands
21. Institute of Experimental hematology and Transfusion Medicine, University Hospital Bonn, Medical Faculty, University of Bonn, Bonn, Germany
22. Molecular Diagnostic Laboratory, CHU Sainte-Justine, Montréal, Québec, Canada
23. Department of Pediatrics, Division of Hematology-Oncology, Montréal University, CHU Sainte-Justine, Montréal, Québec, Canada
24. Department of Medicine, Division of Hematology, University of Calgary, Calgary, Canada
25. Department of Hematology, Radboud university medical center, Nijmegen, the Netherlands
26. Hemophilia Treatment Center Nijmegen-Eindhoven-Maastricht, Nijmegen, the Netherlands
27. Department of Epidemiology and Data Science, Amsterdam Public Health Research Institute, Amsterdam University Medical Centers, University of Amsterdam, the Netherlands

Supplementary Figures

Figure 1. Flow chart for PubMed search assessing for studies reporting on SNPs associated with inhibitor development

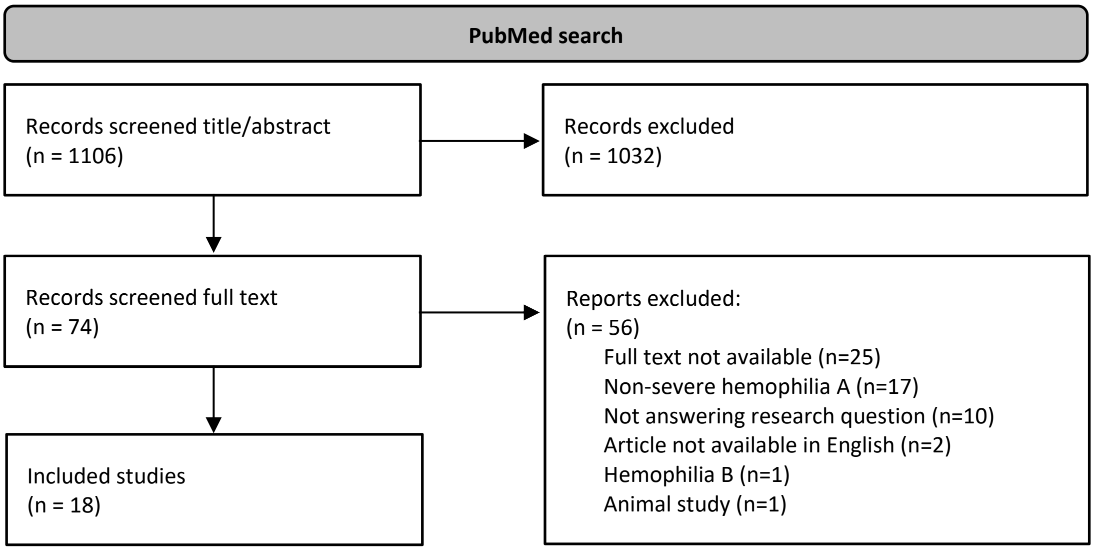

PubMed search terms included (1) severe hemophilia A, (2) congenital or genetics, and (3) inhibitor development.

Figure 2. Flow chart for PubMed search assessing for studies reporting on SNPs in immune checkpoint-related genes associated with immunotherapy outcomes or autoimmune diseases

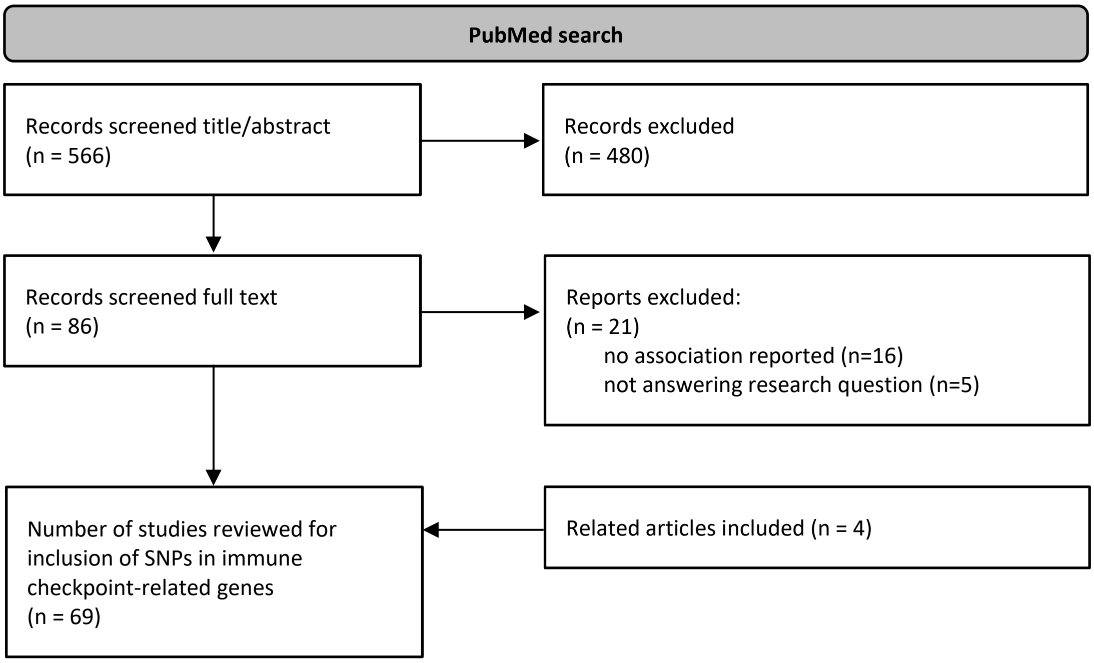

PubMed search terms included (1) immune checkpoint-related genes suggested by Steering Group members, including, GITR, ICOS, PCDC1, PD-L1, TIGIT, HAVCR2, TIM3/TIM-3, LAG3/LAG-3, (2) polymorphism or gene variation, and (3) immune therapy and autoimmune.

Figure 3. Manhattan plot

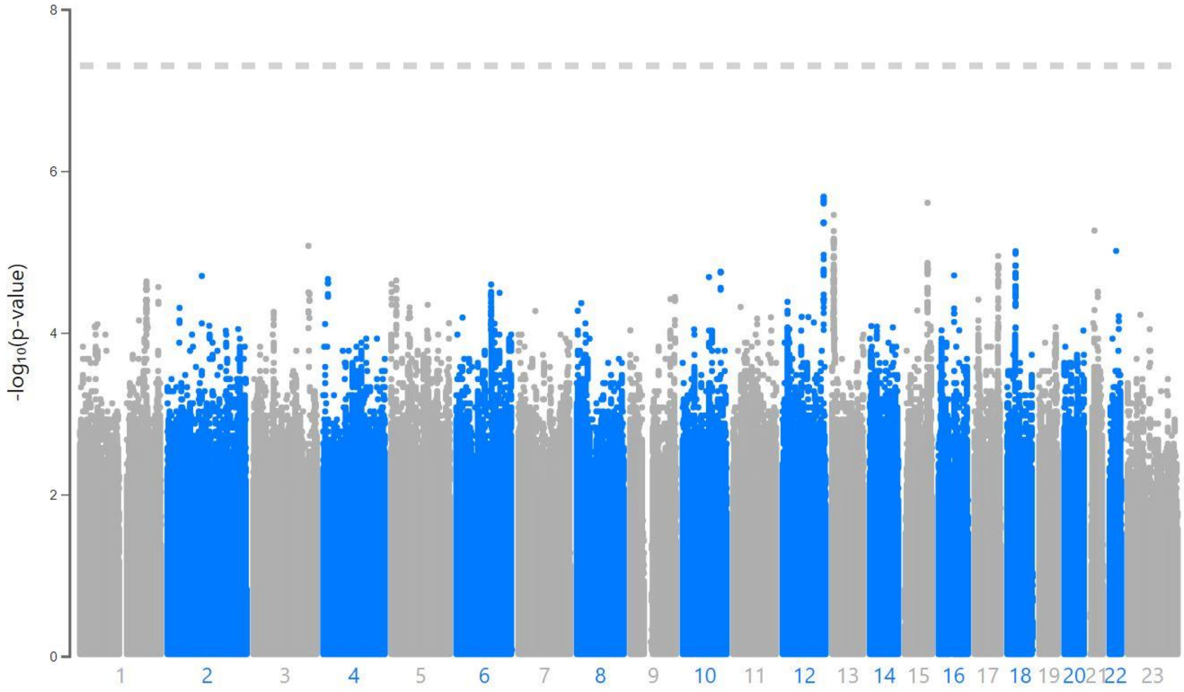

The Manhattan plot shows the association for every variant across the chromosomes. By default, variants with a p-value >0.05 are not plotted. Associations between approximately $1*10^6$ single nucleotide polymorphisms (SNPs) included in the Global Screening Array were assessed for ITI success in this genome-wide association analysis. By correcting for multiple testing by applying the Bonferroni correction, the p-value for significant associations was considered $1*10^{-8}$. Nevertheless, the accepted p-value for suggestive associations was proposed to be below $1*10^{-6}$.[1] None of the p-values of the analyzed SNPs was below the p-value cut-off for suggestive associations.

Figure 4. QQ plot

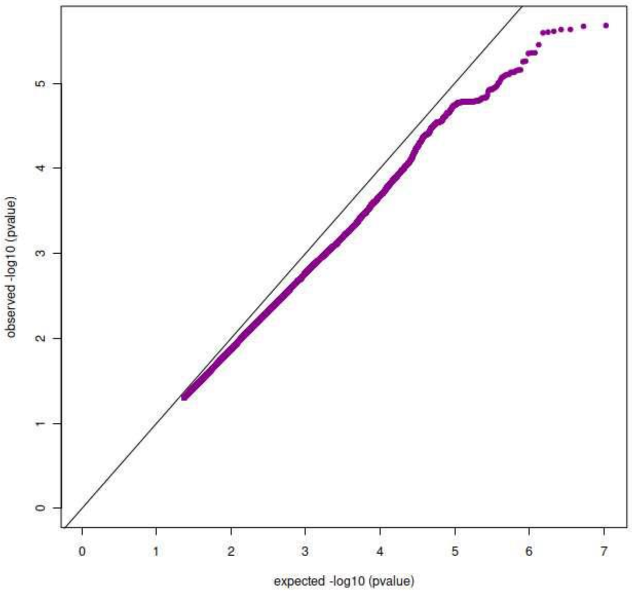

The QQ plot shows that the observed associations are lower than expected by chance, which indicates that our study was underpowered to detect true associations.

Figure 5. Principal component analysis

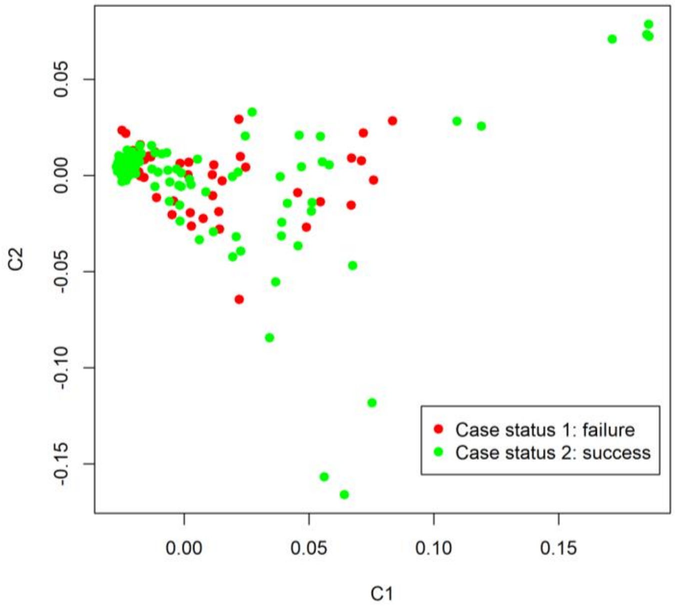

The principal component plot shows the homogeneity of the population. This plot shows the data for component 1 (C1) and 2 (C2). The distribution of cases in this plot is typical for ethnical diversity, with two divergent arms, one for participants with Asian ethnicity, and one for participants with African ethnicity. The red dots represent the persons with ITI failure, the green dots represent persons with ITI success.

Supplementary Tables

Table 1. List of participating Hemophilia Treatment Centers

**Brazil**
Fundação de Hematologia e Hemoterapia de Pernambuco (HEMOPE), Recife
Centro de Hematologia e Hemoterapia do Ceará (HEMOCE), Fortaleza
Centro de Hematologia e Hemoterapia de Santa Catarina (HEMOSC), Florianópolis
Coagulopathy Clinic, Hemocentro do Paraná (HEMEPAR), Curitiba
**Canada**
Hospital for Sick Children, Toronto
McMaster University, Hamilton
CHU Sainte-Justine, Montréal
Foothills Medical Center, Calgary
**The Netherlands**
Radboud university medical center, Nijmegen
Leiden University Medical Center, Leiden
Erasmus University Medical Center, Rotterdam
Amsterdam University Medical Centers, Amsterdam
Van Creveldkliniek, University Medical Center Utrecht, Utrecht
**Germany**
University Bonn, Bonn
**Italy**
IRCCS Humanitas Research Hospital, Rozzano, Milan
Careggi University Hospital, Florence
Federico II University, Naples

Table 2. Candidate SNPs, rationale for inclusion and their effects on ITI success

Gene	rsID	chr	pos	ref	alt	alt_freq	rsq	ß	SE	p-value	rationale	*reference*	LD (r^2)
CD32	rs1801274	1	161479745	A	G	0.518	0.999	-0.349	0.248	0.160	inhibitor development	[2]	
GBP7	rs11804922	1	89596683	A	G	0.045	0.870	-0.159	0.589	0.787	inhibitor development	[3]	
GITR	rs3753348	1	1143657	C	G	0.052	0.843	-0.346	0.553	0.531	steering group + autoimmune disease	[4]	
IGSF2	rs2296449	1	117578750	C	T	0.040	0.971	-0.368	0.597	0.538	inhibitor development	[3]	
IL10	rs1800896	1	206946897	T	C	0.417	1.000	-0.201	0.259	0.438	inhibitor development / *no association found for inhibitor development in one study*	[5-7]	
IL10	rs4072227	1	206957558	C	T	0.910	0.647	0.233	0.509	0.647	inhibitor development	[8]	
IL10	rs4072226	1	206957449	T	C	0.644	0.952	-0.119	0.270	0.659	inhibitor development	[8]	rs6667202 (0.95)
IL10	rs6667202	1	206957092	C	A	0.643	0.992	0.002	0.273	0.995	inhibitor development	[8]	rs4072226 (0.95)
JAK1	rs310247	1	65307409	G	A	0.470	0.957	0.242	0.251	0.335	inhibitor development	[3]	
JAK1	rs2151572	1	65337941	A	C	0.050	0.985	0.023	0.573	0.969	inhibitor development	[3]	
CTLA4	rs4553808	2	204731005	A	G	0.166	1.000	-0.375	0.298	0.209	immune checkpoint-related gene	[9-12]	rs62182595 (0.98), rs16840252 (0.98), rs11571319 (0.98)
CTLA4	rs11571319	2	204738938	G	A	0.164	0.976	-0.325	0.302	0.282	immune checkpoint-related gene	[10, 11]	rs4553808 (0.98), rs16840252 (1.0), rs62182595 (1.0)
CTLA4	rs16840252	2	204731519	C	T	0.166	1.000	-0.375	0.298	0.209	immune checkpoint-related gene	[10, 11]	rs4553808 (0.98), rs62182595 (1.0), rs11571319 (1.0)
CTLA4	rs62182595	2	204731188	G	A	0.165	0.991	-0.374	0.300	0.212	immune checkpoint-related gene	[10]	rs4553808 (0.98), rs16840252 (1.0), rs11571319 (1.0)
CTLA4	rs5742909	2	204732347	C	T	0.090	0.978	-0.418	0.381	0.273	inhibitor development + immune checkpoint-related gene / *no association found for inhibitor development in one study*	[7, 9-11, 13-15]	
CTLA4	rs231775	2	204732714	A	G	0.357	1.000	0.158	0.239	0.508	immune checkpoint-related gene / *no association found for inhibitor development and ITI outcome*	[7, 9, 14-21]	rs11571315 (0.86), rs231779 (0.93)
CTLA4	rs231779	2	204734487	C	T	0.363	0.979	0.122	0.238	0.610	immune checkpoint-related gene	[14]	rs231775 (0.93), rs11571315 (0.88)

CTLA4	rs11571315	2	204730901	T	C	0.567	0.972	-0.162	0.228	0.478	immune checkpoint-related gene	[10, 11]	rs231775 (0.86), rs231779 (0.88)
CTLA4	rs733618	2	204730944	T	C	0.072	0.997	0.290	0.474	0.540	immune checkpoint-related gene	[9-12, 22]	
CTLA4	rs3087243	2	204738919	G	A	0.449	1.000	0.014	0.239	0.955	inhibitor development + immune checkpoint-related gene	[5, 9, 14, 23]	rs11571316 (0.91), rs11571297 (0.87)
CTLA4	rs11571297	2	204745003	T	C	0.487	0.925	0.180	0.243	0.459	immune checkpoint-related gene	[14]	rs3087243 (0.91)
CTLA4	rs11571316	2	204731089	G	A	0.401	1.000	0.034	0.249	0.890	immune checkpoint-related gene	[11]	rs3087243 (0.91)
CTLA4	rs11571317	2	204732008	C	T	0.060	0.703	-0.024	0.649	0.970	immune checkpoint-related gene	[9]	
CTLA4	rs13421616	2	204758695	C	T	0.165	0.925	-0.047	0.312	0.880	immune checkpoint-related gene	[14]	
CTLA4	rs3096849	2	204762957	A	G	0.310	0.961	0.061	0.245	0.803	immune checkpoint-related gene	[14]	
CTLA4	rs10221611	2	204770169	G	A	0.447	0.935	-0.059	0.247	0.810	immune checkpoint-related gene	[14]	
CTLA4	rs2033171	2	204788156	C	T	0.472	0.911	-0.099	0.239	0.678	immune checkpoint-related gene	[14]	rs17268364 (0.88), rs2352551 (0.94)
CTLA4	rs2352551	2	204794757	T	C	0.509	0.866	0.019	0.245	0.938	immune checkpoint-related gene	[14]	rs17268364 (0.97), rs2033171 (0.94)
CTLA4	rs17268364	2	204777818	G	A	0.471	0.917	-0.038	0.241	0.874	immune checkpoint-related gene	[14, 24]	rs2033171 (0.88), rs2352551 (0.97)
DARS1	rs371309040	2	136690425	A	G	0.230	0.923	-0.077	0.299	0.798	inhibitor development	[25]	
DPP4	rs2268894	2	162856148	C	T	0.509	0.958	-0.106	0.232	0.646	inhibitor development	[3]	
ICOS	rs10172036	2	204824283	T	G	0.584	0.781	0.189	0.278	0.496	steering group + immune checkpoint-related gene	[14]	rs10932037 (1.0)
ICOS	rs10932037	2	204825346	C	T	0.090	0.986	-0.179	0.412	0.665	steering group + immune checkpoint-related gene	[26, 27]	rs10932036 (1.0)
ICOS	rs10932029	2	204801768	T	C	0.160	0.695	0.168	0.413	0.683	steering group + immune checkpoint-related gene	[26]	
ICOS	rs6726035	2	205116002	T	C	0.657	0.959	-0.106	0.262	0.685	steering group + immune checkpoint-related gene	[28, 29]	
ICOS	rs117701653	2	204628795	A	C	0.078	0.487	-0.199	0.664	0.765	steering group + immune checkpoint-related gene	[30]	
ICOS	rs11889031	2	204799394	C	T	0.901	0.926	-0.345	0.401	0.389	steering group + immune checkpoint-related gene	[31]	
ICOS	rs4325730	2	204797790	G	A	0.747	1.000	0.268	0.268	0.318	steering group + immune checkpoint-related gene	[32]	
ICOS	rs4521021	2	204816575	T	C	0.207	0.701	0.039	0.359	0.912	steering group + immune checkpoint-related gene	[14]	
ICOS	rs10932036	2	204825181	A	T	0.090	0.987	-0.175	0.412	0.670	steering group +	[14, 33]	

											immune checkpoint-related gene		
ICOS	rs4404254	2	204825286	C	T	0.111	0.931	-0.352	0.369	0.340	steering group + immune checkpoint-related gene	[33, 34]	
ICOS	rs4675379	2	204826095	G	C	0.149	0.975	0.199	0.343	0.562	steering group + immune checkpoint-related gene	[33]	
ICOS	rs10183087	2	204824324	A	C	0.258	0.997	0.147	0.284	0.604	steering group + immune checkpoint-related gene	[34]	
IL1A	rs6746923	2	113553426	A	G	0.618	1.000	-0.329	0.257	0.201	inhibitor development	[8]	
IL1A	rs3783557	2	113530712	C	T	0.233	0.968	-0.330	0.282	0.242	inhibitor development	[8]	
IL1A	rs6716046	2	113564426	T	C	0.238	0.990	0.329	0.298	0.270	inhibitor development	[8]	
IL1A	rs11898680	2	113556867	C	T	0.281	0.984	-0.144	0.253	0.569	inhibitor development	[8]	
IL1A	rs2071373	2	113540084	G	A	0.675	0.959	0.090	0.260	0.729	inhibitor development	[8]	rs7567619 (0.86)
IL1A	rs7567619	2	113564966	T	A	0.335	0.964	-0.124	0.255	0.626	inhibitor development	[8]	rs2071373 (0.86)
IL1A	rs11687624	2	113555592	C	T	0.538	0.997	0.068	0.236	0.773	inhibitor development	[8]	
IL1A	rs12469600	2	113572357	T	C	0.226	0.999	0.064	0.267	0.809	inhibitor development	[8]	
IL1A	rs11680809	2	113570809	C	A	0.570	0.922	-0.060	0.248	0.811	inhibitor development	[8]	
IL1A	rs12711742	2	113571072	C	G	0.873	0.888	-0.061	0.393	0.877	inhibitor development	[8]	rs17597976 (0.85)
IL1A	rs17597976	2	113554636	G	A	0.112	0.964	0.247	0.414	0.551	inhibitor development	[8]	rs12711742 (0.85)
IL1A	rs7585707	2	113569345	T	C	0.304	0.940	0.041	0.265	0.877	inhibitor development	[8]	
IL1ß	rs16944	2	113594867	A	G	0.590	1.000	0.029	0.233	0.903	inhibitor development	[8]	rs1143627 (0.99)
IL1ß	rs1143627	2	113594387	G	A	0.578	1.000	0.070	0.232	0.763	inhibitor development	[8]	rs16944 (0.99)
IL1ß	rs13032029	2	113600415	C	T	0.418	0.981	0.261	0.239	0.275	inhibitor development	[8]	
IL1ß	rs1143623	2	113595829	C	G	0.291	0.989	0.002	0.249	0.995	inhibitor development	[8]	rs13008855 (0.97), rs12053091 (0.91)
IL1ß	rs12053091	2	113610780	T	C	0.293	0.998	-0.019	0.249	0.938	inhibitor development	[8]	rs13008855 (0.93), rs1143623 (0.91)
IL1ß	rs13008855	2	113609568	C	T	0.291	0.989	-0.020	0.251	0.937	inhibitor development	[8]	rs1143623 (0.97), rs12053091 (0.93)
MCM6	rs3087343	2	136622543	T	G	0.238	0.966	-0.030	0.292	0.919	inhibitor development	[25]	rs3754689 (0.92)
LCT	rs3754689	2	136590746	C	T	0.255	0.996	-0.057	0.273	0.835	inhibitor development	[25]	rs3087343 (0.92)
LCT	rs3816155	2	136552526	A	C	0.751	0.923	0.051	0.286	0.858	inhibitor development	[25]	
LCT	rs3213892	2	136552517	G	A	0.232	0.983	-0.069	0.282	0.807	inhibitor development	[25]	
PDCD1	rs10204525	2	242792321	C	T	0.159	0.886	-0.451	0.375	0.229	steering group + immune checkpoint-related gene	[9, 11, 35]	rs41386349 (0.82)
PDCD1	rs41386349	2	242793849	G	A	0.096	0.890	-0.164	0.448	0.714	steering group + immune checkpoint-related gene	[9, 36, 37]	rs10204525 (0.82)

PDCD1	rs7421861	2	242795350	A	G	0.315	0.852	-0.186	0.270	0.492	steering group + immune checkpoint-related gene	[9, 38, 39]	
PDCD1	rs2227982	2	242793433	G	A	0.037	0.998	-0.292	0.608	0.631	steering group + immune checkpoint-related gene + ITI outcome	[9, 11, 16, 35, 36, 40, 41]	rs36084323 (1.0)
PDCD1	rs36084323	2	242801596	C	T	0.036	0.936	-0.290	0.613	0.636	steering group + immune checkpoint-related gene	[9-11, 28, 36-38, 41-45]	rs2227982 (1.0)
PDCD1	rs2227981	2	242793273	A	G	0.598	0.861	-0.046	0.266	0.862	steering group + immune checkpoint-related gene + ITI outcome	[9, 16, 40, 46-51]	
PDCD1	rs11568821	2	242793912	C	T	0.116	0.977	0.036	0.372	0.923	steering group + immune checkpoint-related gene	[9, 21, 38, 48, 52-54]	
PDCD1	rs6710479	2	242798018	T	C	0.530	0.853	-0.031	0.259	0.904	steering group + immune checkpoint-related gene	[39]	
R3HDM1	rs961360	2	136393658	A	G	0.248	0.996	-0.007	0.276	0.980	inhibitor development	[25]	rs12466487 (0.81)
R3HDM1	rs12466487	2	136407078	T	C	0.202	0.909	-0.106	0.311	0.734	inhibitor development	[25]	rs961360 (0.81)
STK17B	rs1519602	2	197027792	T	G	0.620	0.912	0.235	0.269	0.383	inhibitor development	[3]	
UBXN4	rs1050115	2	136511817	A	G	0.223	0.944	-0.073	0.291	0.801	inhibitor development	[25]	
CD86	rs2681401	3	121843127	T	G	0.558	0.790	0.109	0.270	0.687	inhibitor development	[6]	
IL12A	rs2243148	3	159715411	T	C	0.248	0.974	-0.469	0.297	0.114	inhibitor development	[8]	
IL12A	rs2243154	3	159716242	G	A	0.065	0.610	-0.675	0.582	0.246	inhibitor development	[8]	
IL12A	rs2243115	3	159706280	T	G	0.123	0.927	0.431	0.410	0.293	inhibitor development	[8]	
IL12A	rs583911	3	159710390	G	A	0.579	0.950	-0.167	0.241	0.488	inhibitor development	[8]	
IL12A	rs2133310	3	159716352	G	A	0.419	0.981	-0.150	0.238	0.529	inhibitor development	[8]	
IL12A	rs568408	3	159713467	G	A	0.132	0.988	0.068	0.352	0.846	inhibitor development	[8]	
IL12A	rs2243131	3	159712058	A	C	0.163	0.981	0.021	0.311	0.946	inhibitor development	[8]	
TIGIT	rs4682159	3	114028149	G	A	0.200	0.848	-0.053	0.318	0.866	steering group + immune checkpoint-related gene	[40, 55-57]	
IL2	rs2069772	4	123373133	T	C	0.180	0.981	0.092	0.333	0.783	inhibitor development	[8]	
IL2	rs2069762	4	123377980	A	C	0.357	1.000	0.033	0.258	0.899	inhibitor development	[8]	rs4833248 (0.99)
IL2	rs4833248	4	123380405	G	A	0.354	0.984	0.033	0.261	0.899	inhibitor development	[8]	rs2069762 (0.99)
CSF1R	rs1010101	5	149489110	C	T	0.463	0.714	-0.183	0.281	0.516	inhibitor development	[3]	
CSF1R	rs17725712	5	149490808	C	T	0.060	0.984	0.153	0.528	0.772	inhibitor development	[3]	
DOCK2	rs1863993	5	169210180	T	C	0.027	0.685	-0.448	0.775	0.563	inhibitor development	[3]	

HAVCR2	rs1036199	5	156531736	C	A	0.834	1.000	-0.089	0.300	0.767	steering group + immune checkpoint-related gene	[9, 40, 58-62]	rs3087616 (0.94), rs4704846 (0.97), rs4704853 (0.94), rs10515746 (0.94)
HAVCR2	rs3087616	5	156513282	C	T	0.825	0.847	-0.374	0.339	0.270	steering group + immune checkpoint-related gene	[9]	rs4704846 (0.96), rs1036199 (0.94), rs4704853 (0.88), rs10515746 (0.88)
HAVCR2	rs4704846	5	156513344	G	A	0.804	0.853	-0.192	0.314	0.541	steering group + immune checkpoint-related gene	[9]	rs3087616 (0.96), rs1036199 (0.97), rs4704853 (0.97), rs10515746 (0.90)
HAVCR2	rs4704853	5	156536876	A	G	0.791	0.947	0.032	0.289	0.912	steering group + immune checkpoint-related gene	[9]	rs3087616 (0.88), rs1036199 (0.94), rs4704846 (0.97), rs10515746 (1.0)
HAVCR2	rs10515746	5	156536568	A	C	0.791	0.947	0.032	0.289	0.912	steering group + immune checkpoint-related gene	[9, 17, 40, 62, 63]	rs3087616 (0.88), rs1036199 (0.94), rs4704846 (0.90), rs4704853 (1.0)
HAVCR2	rs10053538	5	156537510	C	A	0.055	0.993	0.277	0.568	0.626	steering group + immune checkpoint-related gene	[9, 40, 63]	
IL13	rs20541	5	131995964	A	G	0.776	0.999	-0.686	0.334	0.040	inhibitor development	[3]	
IQGAP2	rs17652304	5	75877193	C	G	0.043	0.845	-0.835	0.584	0.153	inhibitor development	[3]	
MAPK9	rs4147385	5	179677517	G	A	0.252	0.807	0.467	0.327	0.153	inhibitor development	[3]	
PDGFRB	rs10072056	5	149505306	G	A	0.320	0.947	-0.179	0.261	0.491	inhibitor development	[3]	
PDGFRB	rs1075846	5	149504158	T	C	0.393	0.898	-0.141	0.271	0.602	inhibitor development	[3]	
F13A1	rs13206518	6	6153002	T	C	0.046	0.785	-0.017	0.656	0.979	inhibitor development	[3]	
IL1ß	rs1261220	6	126597654	G	A	0.189	0.981	0.629	0.366	0.086	inhibitor development	[8]	
PTPRK	rs4498385	6	128450019	T	G	0.086	0.580	0.052	0.545	0.923	inhibitor development	[3]	
PTPRK	rs2179694	6	128363723	G	C	0.123	0.816	0.018	0.421	0.966	inhibitor development	[3]	
STXBP5	rs9390459	6	147680359	A	G	0.544	1.000	0.070	0.244	0.775	plasma level VWF	[64, 65]	
TNFA	rs1799724	6	31542482	C	T	0.122	1.000	0.382	0.384	0.320	inhibitor development	[7]	
TNFA	rs1800629	6	31543031	G	A	0.147	1.000	0.112	0.361	0.757	inhibitor development / *no association found for inhibitor development in one study*	[5, 7]	

TNFRSF21	rs10807350	6	47228912	G	A	0.878	0.457	0.827	0.495	0.095	inhibitor development	[3]	
PTPRN2	rs12667537	7	157650340	G	T	0.572	0.885	-0.038	0.268	0.888	inhibitor development	[3]	
IDO1	rs7820268	8	39777529	C	T	0.306	0.995	-0.295	0.253	0.243	steering group + autoimmune disease	[66]	rs10108662 (0.95)
IDO1	rs10108662	8	39779989	C	A	0.297	0.923	-0.286	0.265	0.281	steering group + altered enzyme activity	[67]	rs7820268 (0.95)
IDO1	rs3739319	8	39785321	G	A	0.326	0.925	0.221	0.266	0.406	steering group + altered enzyme activity	[67]	
IDO1	rs9298586	8	39782407	T	G	0.038	0.734	0.568	0.728	0.435	steering group + altered enzyme activity	[67]	
MSR1	rs1564819	8	16005211	G	C	0.371	0.839	-0.078	0.273	0.775	Inhibitor development	[3]	
SCARA5	rs2726953	8	27801305	G	A	0.295	0.898	0.360	0.285	0.205	plasma level VWF	[64, 65]	
SCARA5	rs9644133	8	27814483	C	T	0.183	0.900	-0.269	0.305	0.378	plasma level FVIII	[64]	rs11780263 (0.98)
SCARA5	rs11780263	8	27823832	G	A	0.190	0.993	-0.280	0.281	0.319	plasma level FVIII	[64]	rs9644133 (0.98)
ABO	rs7853989	9	136131592	G	C	0.090	1.000	-0.335	0.399	0.402	pharmacokinetics rFVIII	[68]	
ABO	rs687289	9	136137106	G	A	0.359	1.000	-0.002	0.235	0.994	plasma level FVIII	[64]	rs687621 (0.98)
ABO	rs687621	9	136137065	A	G	0.359	1.000	-0.002	0.235	0.994	plasma level VWF	[64]	rs687289 (0.98)
PD-L1	rs2297136	9	5467955	G	A	0.574	0.716	0.036	0.256	0.889	steering group + immune checkpoint-related gene + ITI outcome	[16, 69]	
PD-L1	rs2890658	9	5465130	C	A	0.118	0.730	-0.317	0.458	0.489	steering group + immune checkpoint-related gene	[9, 70]	
PD-L1	rs4742098	9	5470497	A	G	0.215	0.908	0.195	0.306	0.524	steering group + immune checkpoint-related gene	[9]	
PD-L1	rs4143815	9	5468257	G	C	0.259	0.853	-0.179	0.285	0.531	steering group + immune checkpoint-related gene	[9, 38, 71-75]	
PD-L1	rs10815225	9	5450497	G	C	0.141	0.931	0.192	0.362	0.596	steering group + immune checkpoint-related gene	[9, 72]	
PD-L1	rs866066	9	5450953	C	T	0.415	0.915	0.100	0.254	0.693	steering group + immune checkpoint-related gene	[9]	
PD-L1	rs2890657	9	5452560	G	C	0.204	0.943	-0.031	0.303	0.919	steering group + immune checkpoint-related gene	[9]	
PD-L1	rs822338	9	5451557	C	T	0.720	0.901	0.017	0.290	0.953	steering group + immune checkpoint-related gene	[9]	rs822339 (0.95), rs1411262 (0.94)
PD-L1	rs822339	9	5453172	A	G	0.720	0.902	0.014	0.289	0.960	steering group + immune checkpoint-related gene	[76]	rs822338 (0.95), rs1411262 (0.96)

PD-L1	rs1411262	9	5459419	C	T	0.283	0.999	0.076	0.275	0.782	steering group + immune checkpoint-related gene	[76, 77]	rs822338 (0.94), rs822339 (0.96)
PD-L1	rs822336	9	5448690	G	C	0.474	0.938	-0.071	0.247	0.775	steering group + immune checkpoint-related gene	[78]	
PD-L1	rs822335	9	5448218	T	C	0.636	0.995	-0.007	0.251	0.979	steering group + immune checkpoint-related gene	[79]	
PTPRD	rs10977313	9	8828849	G	T	0.121	0.594	-0.539	0.444	0.225	inhibitor development	[3]	
MBL2	rs1800451	10	54531226	C	T	0.030	0.999	0.259	0.738	0.725	inhibitor development	[80]	
MBL2	rs1800450	10	54531235	C	T	0.152	0.992	-0.116	0.342	0.734	inhibitor development	[80]	
MBL2	rs5030737	10	54531242	G	A	0.064	0.995	-0.125	0.477	0.793	inhibitor development + plasma levels MBL	[80, 81]	
PTPRE	rs7099752	10	129751331	G	A	0.154	0.673	0.428	0.434	0.325	inhibitor development	[3]	
PTPRE	rs11015985	10	129779318	C	T	0.173	0.910	0.119	0.343	0.729	inhibitor development	[3]	
-	rs2509897	11	57362617	C	G	0.640	0.932	-0.206	0.249	0.408	inhibitor development	[3]	
CD44	rs10836342	11	35241637	C	G	0.310	0.988	0.540	0.282	0.056	inhibitor development	[3]	
CD44	rs927335	11	35250518	A	T	0.346	0.942	0.317	0.266	0.234	inhibitor development	[3]	
SRPRA	rs638766	11	126134698	T	C	0.748	0.964	0.583	0.273	0.033	inhibitor development	[3]	
ST3GAL4	rs2186717	11	126261832	G	A	0.515	0.956	-0.152	0.259	0.557	plasma level VWF	[65]	
HSP90B1	rs1882019	12	104337667	G	A	0.092	0.918	0.123	0.451	0.785	inhibitor development	[3]	
INFG	rs2430561	12	68552522	T	A	0.463	1.000	-0.003	0.247	0.990	inhibitor development	[82]	
LAG-3	rs2365094	12	6883670	G	C	0.335	0.488	-0.258	0.351	0.461	steering group + immune checkpoint-related gene	[9]	
LAG-3	rs3782735	12	6885076	G	A	0.55	0.964	-0.122	0.235	0.605	steering group + immune checkpoint-related gene	[9, 40]	
LAG-3	rs870849	12	6887020	T	C	0.552	0.970	0.016	0.241	0.947	steering group + immune checkpoint-related gene	[28, 40, 42]	
LRP1	rs34577247	12	57578673	G	A	0.016	0.998	-0.111	0.893	0.901	plasma level VWF	[65]	
LRP1	rs1800127	12	57539082	C	T	0.016	0.727	-0.093	1.224	0.940	plasma level VWF	[65]	
MLL2	rs10875912	12	49416944	T	C	0.334	0.994	0.088	0.256	0.732	inhibitor development	[3]	
OASL	rs10849829	12	121470256	G	A	0.465	0.964	0.021	0.240	0.930	inhibitor development	[3]	
PLXNC1	rs870034	12	94640095	T	C	0.525	0.991	-0.193	0.246	0.432	inhibitor development	[3]	
STAB2	rs4981022	12	104149874	G	A	0.655	0.898	0.117	0.265	0.660	steering group + plasma level VWF	[64, 83]	
STAB2	rs12229292	12	104153633	G	T	0.244	0.908	-0.046	0.284	0.871	steering group + plasma level FVIII	[83]	
STX2	rs7978987	12	131281494	A	G	0.300	0.793	-0.370	0.268	0.169	plasma level VWF	[64, 65]	

VWF	rs1063856	12	6153534	T	C	0.327	0.854	-0.115	0.286	0.687	plasma level FVIII	[64]	rs1063857 (0.99)
VWF	rs1063857	12	6153514	A	G	0.327	0.854	-0.114	0.286	0.690	plasma level VWF	[64]	rs1063856 (0.99)
ALOX5AP	rs4075131	13	31310919	A	G	0.265	0.937	0.303	0.281	0.280	inhibitor development	[3]	
BAFF	rs9514828	13	108921373	C	T	0.392	0.630	0.307	0.305	0.313	inhibitor development	[84]	
TC2N	rs10133762	14	92292769	T	G	0.533	0.940	0.310	0.246	0.208	plasma level VWF	[64, 65]	
WDR20	rs1190587	14	102608093	T	C	0.815	0.741	-0.492	0.346	0.155	inhibitor development	[3]	
BLM	rs414634	15	91356253	A	C	0.684	0.996	0.251	0.254	0.325	inhibitor development	[3]	
IGF1R	rs4966019	15	99274326	C	T	0.589	0.941	0.051	0.230	0.825	inhibitor development	[3]	
IGF1R	rs3803476	15	99256570	A	G	0.624	0.752	0.003	0.265	0.992	inhibitor development	[3]	
IRF8	rs1044873	16	85955671	C	T	0.379	0.996	0.231	0.245	0.347	inhibitor development	[3]	
LITAF	rs11646660	16	11648180	C	T	0.096	0.512	-0.450	0.496	0.364	inhibitor development	[3]	
C17orf85	rs17763453	17	3718135	T	C	0.156	0.439	-0.902	0.457	0.048	inhibitor development	[3]	
MAP2K4	rs3826392	17	11922904	G	T	0.765	0.999	0.071	0.263	0.786	inhibitor development	[3]	rs12051769 (0.94), rs12325842 (0.86)
MAP2K4	rs12051769	17	11977952	G	A	0.729	0.826	0.087	0.280	0.756	inhibitor development	[3]	rs3826392 (0.94), rs12325842 (0.90)
MAP2K4	rs12325842	17	11994772	A	T	0.181	0.877	0.015	0.321	0.963	inhibitor development	[3]	rs12051769 (0.90), rs3826392 (0.86)
PCGF2	rs2879097	17	36889559	C	T	0.215	0.891	0.200	0.314	0.524	inhibitor development	[3]	
-	rs11152093	18	56418253	G	A	0.457	0.961	0.082	0.250	0.744	inhibitor development	[3]	
CD226	rs1007822	18	67555221	C	T	0.463	0.999	-0.323	0.239	0.176	inhibitor development	[3]	rs12969613 (0.98)
CD226	rs12969613	18	67559432	A	T	0.456	0.971	-0.300	0.241	0.213	inhibitor development	[3]	rs1007822 (0.98)
CD226	rs10513983	18	67552338	T	C	0.702	0.937	0.156	0.274	0.568	inhibitor development	[3]	
MAPK4	rs12959952	18	48240837	C	T	0.338	0.964	-0.094	0.261	0.719	inhibitor development	[3]	
PTPRM	rs8086815	18	8173669	G	A	0.123	0.915	-0.526	0.353	0.136	inhibitor development	[3]	
TFGB1	rs1982037	18	38434784	C	T	0.136	0.650	-0.070	0.424	0.870	inhibitor development	[82]	
CLEC4M	rs868875	19	7831166	A	G	0.336	0.988	0.031	0.253	0.902	pharmacokinetics rFVIII + plasma level VWF	[64, 68, 85, 86]	
FUT2	rs601338	19	49206674	G	A	0.423	0.989	-0.096	0.241	0.689	plasma level VWF	[65]	
PKIG	rs244090	20	43234090	A	G	0.180	0.898	-0.292	0.318	0.358	inhibitor development	[3]	
AIRE	rs1003854	21	45710107	T	C	0.235	0.731	0.105	0.345	0.760	inhibitor development	[3]	
ECGF1	rs470119	22	50966914	T	C	0.635	0.995	-0.154	0.238	0.517	inhibitor development	[3]	
HMOX1	rs2071749	22	35783413	A	G	0.558	0.695	0.122	0.278	0.662	steering group + inhibitor development	[87]	
HMOX1	rs2071746	22	35776672	A	T	0.446	0.791	0.108	0.268	0.687	steering group + inhibitor development	[87]	

YWHAH	rs7290696	22	32341684	C	T	0.181	0.826	0.661	0.382	0.084	inhibitor development	[3]
F8	rs5945269	X	154219318	A	G	0.116	0.580	0.337	0.213	0.115	inhibitor development	[8]
F8	rs6649625	X	154248283	G	A	0.321	0.418	-0.142	0.138	0.304	inhibitor development	[8]
F8	rs6643622	X	154173530	G	T	0.610	0.566	-0.092	0.116	0.429	inhibitor development	[8]
F8	rs4898399	X	154087368	T	A	0.784	0.670	0.090	0.117	0.442	inhibitor development	[8]
F8	rs5945270	X	154223203	C	T	0.025	0.376	0.404	0.530	0.446	inhibitor development	[8]
F8	rs1936645	X	154198170	G	C	0.293	0.744	0.079	0.108	0.468	inhibitor development	[8]
F8	rs17281398	X	154233774	A	G	0.089	0.459	0.103	0.238	0.666	inhibitor development	[8]
FOXP3	rs56066773	X	49107623	G	A	0.023	0.309	-0.585	0.668	0.381	steering group + autoimmune disease	[88]
FOXP3	rs2232367	X	49113312	G	A	0.021	0.589	-0.033	0.338	0.921	steering group + autoimmune disease	[88]
FOXP3	rs2294020	X	49103224	G	A	0.367	0.963	-0.106	0.089	0.232	steering group + autoimmune disease	[89]

Abbreviations. rsID = unique label to identify a specific single nucleotide polymorphism, chr = chromosome, pos = position on chromosome, ref = reference allele, alt = alternative allele, alt-freq = frequency alternative allele, rsq = estimated value of the squared correlation between imputed genotypes and observed genotypes, ß = effect estimate, SE = standard error, LD = linkage disequilibrium, ITI = immune tolerance induction, VWF = von Willebrand factor. Results are sorted by chromosome numbers. This table shows the position of each SNP, together with the (nearest) gene. The reference allele is presented, together with the alternative allele and its frequency (alt_freq). The rsq presents the imputation quality, ranging from 0 (poor imputation) to 1 (optimal imputation). A rsq > 0.8 is good, between 0.3 and 0.8 is intermediate (marked in red), and data with rsq < 0.3 were removed due to poor imputation quality. If an genetic variation was in LD with another variant, this is presented in the most right column.

Table 3. Candidate HLA-variants, rationale for inclusion and their effects on ITI success

Locus	ß	SE	p-value	rationale	*reference*
**HLA-class I**					
A3	0,740	0,425	0,082	inhibitor development	[90]
B7	0,641	0,532	0,228	inhibitor development	[90]
C7	0,182	0,284	0,522	inhibitor development	[90]
C16	-0,936	0,604	0,122	inhibitor development	[91]
**HLA-class II**					
DRB1*01	0,370	0,445	0,406	inhibitor development	[92]
DRB1*11	0,236	0,420	0,574	inhibitor development	[92]
DRB1*11:01	0,113	0,597	0,849	inhibitor development	[92]
DRB1*11:04	0,790	0,924	0,392	inhibitor development	[93]
DRB1*14	0,682	0,741	0,357	inhibitor development	[91]
DRB1*15	0,016	0,324	0,961	inhibitor development	[5, 6]
DQA1*01:02	0,065	0,311	0,835	inhibitor development	[90, 94]
DQB1*03	0,338	0,242	0,163	inhibitor development	[92]
DQB1*03:01	0,271	0,296	0,360	inhibitor development	[92]
DQB1*05:01	-0,088	0,373	0,814	inhibitor development	[92]
DQB1*06:02	0,190	0,388	0,624	inhibitor development	[5, 90]
DPB1*02:02	-0,373	0,298	0,210	inhibitor development	[93]

Abbreviation. ß = effect estimate, SE = standard error.

Table 4. SNPs not covered by the GSA BeadChip, however, were previously associated with inhibitor development, ITI outcome or steering group suggested genes.

**Gene**	**rsID**	**chr**	**pos**	**previously reported association**	***reference***
HSPG2	rs2270699	1	22161304	immune checkpoint-related gene + autoimmune disease	[95]
IL1ß	rs6735738	1	224626125	inhibitor development	[8]
BLTA	rs16859633	1	112198335	immune checkpoint-related gene	[9]
BLTA	rs2931761	1	112190137	immune checkpoint-related gene	[9]
PDCD1	rs5839828	2	242801753	immune checkpoint-related gene	[11]
HAVCR2	rs891246256	5	156537535	immune checkpoint-related gene	[9]
IDO1	rs35099072	8	39775653	autoimmune disease	[66]
IDO1	rs35059413	8	39771451	altered enzyme activity	[67]
IDO1	rs61753677	8	39777621	altered enzyme activity	[67]
PD-L1	rs17718883	9	5462876	immune checkpoint-related gene	[9, 38, 96]
STAB2	rs141041254	12	104152932	plasma level VWF and FVIII	[83]
UFM1	rs17057285	13	38737821	plasma level VWF	[65]
FUT1	rs104894686	13	49253591	plasma level VWF	[65]
F8	rs5945250	X	154059412	inhibitor development	[8]
F8	rs17281377	X	154066470	inhibitor development	[8]
F8	rs7053448	X	154193211	inhibitor development	[8]
FOXP3	rs6609859	X	49119666	autoimmune disease	[88]
FOXP3	rs2232368	X	49112283	autoimmune disease	[88]
FOXP3	rs11465476	X	49110323	autoimmune disease	[88]
FOXP3	rs148013438	X	49107122	autoimmune disease	[88]
FOXP3	rs3761548	X	49118241	autoimmune disease	[88]

Abbreviations. rsID = unique label to identify a specific single nucleotide polymorphism, chr = chromosome, pos = position on chromosome.

Table 5. SNPs that were previously reported not to be associated with inhibitor development or ITI outcome and therefore not selected as candidate predictors in the current study.

Gene	rsID	chr	pos	ref	alt	alt_freq	rsq	ß	SE	p-value	*reference*
IL10	rs1800871	1	206946634	A	G	0.730	1.000	-0.425	0.291	0.144	[7]
IL10	rs1800872	1	206946407	T	G	0.730	1.000	-0.425	0.291	0.144	[7]
IL1ß	rs1143634	2	113590390	G	A	0.222	0.992	0.149	0.292	0.611	[7]
MCM6	rs4988235	2	136608646	G	A	0.399	0.998	-0.154	0.253	0.541	[25]
PDCD1	rs370111035	2	242800952*								[16]
IL4	rs2243250	5	132009154	C	T	0.205	0.997	0.236	0.332	0.478	[7]
TNFA	rs1800630	6	31542476	C	A	0.184	1.000	0.579	0.337	0.086	[7]
TNFA	rs361525	6	31543101	G	A	0.041	1.000	-1.151	0.559	0.040	[7]
TNFA	rs3093662	6	31544189	A	G	0.058	1.000	-0.802	0.451	0.075	[7]
TNFA	rs4248158	6	31542533	C	T	0.023	1.000	0.307	0.849	0.718	[7]
TNFA	rs1799724	6	31542482	C	T	0.122	1.000	0.382	0.384	0.320	[7]

Abbreviations. rsID = unique label to identify a specific single nucleotide polymorphism, chr = chromosome, pos = position on chromosome, ref = reference allele, alt = alternative allele, alt-freq = frequency alternative allele, rsq = estimated value of the squared correlation between imputed genotypes and observed genotypes, ß = effect estimate, SE = standard error. *SNP not covered by the GSA BeadChip.

Table 6. Exclusion of participants in a subset of genetic analysis.

PID	Reason for exclusion	ITI outcome	Reported ethnicity	*F8* mutation type	Age at inhibitor development (months)	Inhibitor titer at detection (BU/mL)	Pre-ITI titer (BU/mL)	Peak inhibitor titer ever measured (BU/mL)	Age at ITI start (months)	FVIII product at ITI start	ITI dose at ITI start (IU/kg/day)
**GSA/GWAS**											
114	Sample swap causing identical genetic data with another person.	Success	Asian	Int. 22 inv.	11.7	1.6	6.4	1331.2	25.6	rFVIII	200
209	Poor quality DNA sample.	Failure	Arab/Middle Eastern	Int. 22 inv.	25.7	0.5	220.0	520.0	29.3	rFVIII	90
701	No IC for GSA.	Failure	Caucasian	Unknown	24.0	6.5	7.2	72.8	28.2	rFVIII	20
702	No IC for GSA.	Success	Caucasian	Int. 22 inv.	3.3	16.3	9.2	73.6	8.2	rFVIII	18
703	No IC for GSA.	Success	Caucasian	Int. 22 inv.	17.8	18.7	unknown	224.0	30.5	rFVIII	13
704	No IC for GSA.	Success	Caucasian	Unknown	39.1	4.8	4.8	4.8	39.6	rFVIII	13
1002	Sample swap causing identical genetic data with another person.	Success	Mixed	Int. 22 inv.	5.2	2.4	3.6	3.6	5.3	rFVIII	21
1007	Insufficient amount of DNA.	Success	Caucasian	Int. 1 inv.	13.5	107.0	11.3	1000.0	26.1	rFVIII	21
***FCGR2/3* gene locus variation**											
209	Poor quality DNA sample.	Failure	Arab/Middle Eastern	Int. 22 inv.	25.7	0.5	220.0	520.0	29.3	rFVIII	90
1639	Insufficient amount of DNA.	Failure	Hispanic/Latino	Missense mutation	33.2	1.3	2.2	40.0	94.2	pdFVIII	21

Abbreviations. PID = patient study ID, GSA = global screening array, MLPA = multiplex ligation-dependent probe amplification, IC = informed consent, Int. 22 inv. = intron 22 inversion, Int 1 inv = intron 1 inversion, ITI = immune tolerance induction, BU = Bethesda Units, IU = international units, FVIII = factor VIII, rFVIII = recombinant FVIII, pdFVIII = plasma-derived FVIII.

Table 7. Logistic regression analysis of polymorphism in IL-10 promoter region (CA short tandem repeats) on ITI success in the total population, with or without correction for genetic principal components.

	**Success** (N=147)		**Failure** (N=57)		**Fisher's Exact**	**Single variant, unadjusted**			**Single variant, number of ref. allele, adjusted PC 1-5***			**Single variant, number of ref. allele, adjusted PC 1-3***		
**Alleles**	**1**	**2**	**1**	**2**	**p-value**	**OR**	**95% CI**	**p-value**	**OR**	**95% CI**	**p-value**	**OR**	**95% CI**	**p-value**
**CA repeats per allele**														
17	1	0	0	0	1.000	n.a.	0.00-inf	0.988	n.a.	0.00-inf	0.988	n.a.	0.00-inf	0.988
18	0	0	0	0	n.a.	n.a.	n.a.	n.a.	n.a.	n.a.	n.a.	n.a.	n.a.	n.a.
19	8	0	2	0	0.729	1.58	0.33-7.69	0.569	46.8	$0.10\text{-}2.15*10^4$	0.219	5.13	0.51-51.13	0.164
20	17	1	6	0	1.000	1.24	0.49-3.16	0.654	1.17	0.42-3.25	0.766	1.33	0.49-3.64	0.575
21	67	18	26	5	0.794	1.17	0.73-1.87	0.506	1.28	0.74-2.19	0.376	1.10	0.67-1.81	0.695
22	16	1	9	1	0.340	0.63	0.30-1.35	0.235	0.59	0.26-1.31	0.194	0.63	0.29-1.41	0.263
23	33	2	12	1	1.000	1.03	0.53-1.99	0.933	0.97	0.48-1.96	0.923	1.03	0.52-2.03	0.935
24	19	1	10	0	0.635	0.80	0.36-1.76	0.576	0.71	0.30-1.69	0.442	0.81	0.35-1.88	0.625
25	45	6	15	3	0.755	1.06	0.62-1.83	0.828	1.08	0.60-1.96	0.795	1.10	0.62-1.97	0.746
26	25	0	11	0	0.687	0.86	0.39-1.88	0.700	0.72	0.31-1.66	0.436	0.72	0.32-1.61	0.422
27	4	0	2	0	0.673	0.77	0.14-4.32	0.766	0.88	0.14-5.42	0.894	0.86	0.14-5.34	0.874
28	1	0	0	0	1.000	n.a.	0.00-inf	0.988	n.a.	0.00-inf	0.987	n.a.	0.00-inf	0.987
29	0	0	1	0	0.279	0.00	0.00-inf	0.986	0.00	0.00-inf	0.986	0.00	0.00-inf	0.986
**Large number of CA repeats**														
≥22	73	45	26	22	0.531	0.78	0.50-1.22	0.278	0.68	0.41-1.12	0.127	0.76	0.48-1.22	0.257
≥23	71	37	27	16	0.936	0.91	0.60-1.39	0.665	0.84	0.52-1.34	0.462	0.89	0.57-1.40	0.621
≥24	64	22	27	9	0.867	0.90	0.58-1.38	0.617	0.85	0.53-1.36	0.492	0.88	0.56-1.38	0.575
≥25	65	11	23	6	0.752	0.95	0.59-1.53	0.824	0.92	0.54-1.56	0.752	0.92	0.55-1.52	0.733
≥26	30	0	14	0	0.570	0.79	0.38-1.62	0.518	0.72	0.33-1.57	0.409	0.69	0.33-1.47	0.341
≥27	5	0	3	0	0.688	0.63	0.15-2.74	0.542	0.84	0.18-3.95	0.825	0.71	0.15-3.28	0.658
**Sum of alleles**														
Continuous						0.95	0.86-1.06	0.354	0.93	0.82-1.04	0.199	0.94	0.84-1.05	0.245

Abbreviations. N = total number of participants, OR = odds ratio, CI = confidence interval, ref = reference, PC = principal component. *These data are without 8 patients of whom PCA data were lacking.

Table 8. Logistic regression analysis of *FCGR* variations on ITI success in total population, with or without correction for genetic principal components.

Genotype	Total cohort (N = 202) n (%N)	Success (n=147) n (%)	Failure (n=55) n (%)	Single variant, unadjusted			Number of ref. allele[‡]	Single variant, adjusted PC 1-5[*]			Single variant, adjusted PC 1-3[*]		
				OR	95% CI	p-value		OR	95% CI	p-value	OR	95% CI	p-value
**FCGR2A (p.His166Arg)**													
HH	46 (22.8)	35 (76.1)	11 (23.9)	*ref*	*ref*	*ref*	H	1.29	0.79-2.12	0.311	1.30	0.81-2.09	0.282
HR	105 (52.0)	77 (73.3)	28 (26.7)	0.86	0.39-1.93	0.722							
RR	51 (25.2)	35 (68.6)	16 (31.4)	0.69	0.28-1.69	0.414							
**FCGR2A (p.Gln62Trp)**													
QQ	163 (80.7)	120 (73.6)	43 (26.4)	*ref*	*ref*	*ref*	W	0.83	0.38-1.83	0.645	0.91	0.42-1.95	0.802
QW	38 (18.8)	26 (68.4)	12 (31.6)	0.78	0.36-1.67	0.518							
WW	1 (0.5)	1 (100.0)	0 (0.0)	n.a.	n.a.	n.a.							
**FCGR2B (p.Ile232Thr)**													
II	147 (72.8)	106 (72.1)	41 (27.9)	*ref*	*ref*	*ref*	T	1.01	0.49-2.08	0.980	1.28	0.64-2.54	0.481
IT	52 (25.7)	38 (73.1)	14 (26.9)	1.05	0.52-1.14	0.893							
TT	3 (1.5)	3 (100.0)	0 (0.0)	n.a.	n.a.	n.a.							
**FCGR2C (p.Gln57Ter)[§]**													
STOP	149 (73.8)	107 (71.8)	42 (28.2)	*ref*	*ref*	*ref*	STOP	1.09	0.65-1.81	0.749	0.95	0.58-1.57	0.853
ORF (w/STOP or ncORF)	44 (21.8)	32 (72.7)	12 (27.3)	1.05	0.49-2.22	0.905	ORF	0.91	0.41-2.03	0.825	0.99	0.45-2.14	0.970
ncORF (w/STOP)	7 (3.5)	6 (85.7)	1 (14.3)	2.36	0.28-20.16	0.434	ncORF	0.84	0.33-2.12	0.713	1.02	0.41-2.57	0.963
Missing allele	*2 (2.0)*	*2 (100.0)*	*0 (0.0)*										
**FCGR3A (p.Val176Phe)**													
V[†]	27 (13.4)	19 (70.4)	8 (29.6)	*ref*	*ref*	*ref*	V	0.79	0.48-1.31	0.371	0.93	0.58-1.49	0.760
VF	90 (44.6)	65 (72.2)	25 (27.8)	1.10	0.43-2.82	0.851							
F[†]	85 (42.1)	63 (74.1)	22 (25.9)	1.21	0.46-3.14	0.702							
**FCGR2B promoter haplotypes**													
2B.1	167 (82.7)	121 (72.5)	46 (27.5)	*ref*	*ref*	*ref*	2B.4	1.22	0.48-3.07	0.678	1.19	0.50-2.82	0.693
2B.1/2B.2	1 (0.5)	0 (0.0)	1 (100.0)	n.a.	n.a.	n.a.							
2B.1/2B.4	33 (16.3)	25 (75.8)	8 (24.2))	1.19	0.50-2.82	0.696							
2B.4	1 (0.5)	1 (100.0)	0 (0.0)	n.a.	n.a.	n.a.							
**FCGR2C promoter haplotypes**													
2B.1	156 (77.2)	113 (72.4)	43 (27.6)	*ref*	*ref*	*ref*	2B.2	0.91	0.41-2.03	0.825	0.99	0.45-2.14	0.970
2B.1/2B.2	43 (21.3)	31 (72.1)	12 (27.9)	0.96	0.46-2.09	0.964							

2B.2	1 (0.5)	1 (100.0)	0 (0.0)	n.a.	n.a.	n.a.							
Missing or missing allele	*2 (1.0)*	*2 (100.0)*	*0 (0.0)*										
**FCGR3B haplotypes**													
NA1	32 (15.8)	23 (71.9)	9 (28.1)	*ref*	*ref*	*ref*	NA1	1.01	0.61-1.68	0.971	1.02	0.62-1.65	0.951
NA1/NA2	95 (47.0)	71 (74.7)	24 (25.3)	1.16	0.47-2.84	0.750	NA2	0.95	0.59-1.54	0.847	0.93	0.59-1.47	0.758
NA2	74 (36.6)	52 (70.3)	22 (29.7)	0.93	0.37-2.32	0.868							
Missing or missing allele	*2 (1.0)*	*1 (100.0)*	*0 (0.0)*										

Abbreviations. N = total number of participants, OR = odds ratio, CI = confidence interval, ref = reference, PC = principal component, ORF = open reading frame, ncORF = nonclassic ORF, w/ = with. [†]Allele variation between one or two V or F. [‡]This column shows the reference allele of the logistic regression data, which presents the effect of each number of reference allele on ITI success. [*]These data are without 8 patients of whom PCA data were lacking. [§]Combinations for this SNP were reported as follows: having an ORF allele was dominant, thus people with an ORF allele, with or without a STOP or ncORF allele, were reported under 'ORF (w/STOP or ncORF)'. People with a ncORF allele, with or without a STOP allele were reported under 'ncORF (w/STOP)'. People with only STOP allele(s) were reported under 'STOP'. The combinations for this SNP included: for ORF (ORF (n=1), ORF/STOP (n=36), ORF/STOP/STOP (n=4), ORF/ncORF (n=1), ORF/ncORF/ncORF (n=2)); for ncORF (ncORF (n=2), ncORF/STOP (n=1), ncORF/STOP/STOP (n=1), ncORF/ncORF/STOP (n=2), ncORF/ncORF/STOP/STOP (n=1)); for STOP (STOP (n=19), STOP/STOP (n=119), STOP/STOP/STOP (n=10), STOP/STOP/STOP/STOP (n=1)).

Table 9. Logistic regression analysis of polymorphism in IL-10 promoter region (CA short tandem repeats) in a genetically homogenous European-ancestry population, with or without correction for genetic principal components.

	**Success** (N=92)		**Failure** (N=21)		**Fisher's Exact**	**Single variant, unadjusted**			**Single variant, number of ref. allele, adjusted PC1-5***			**Single variant, number of ref. allele, adjusted PC1-3***		
**Alleles**	**1**	**2**	**1**	**2**	**p-value**	**OR**	**95% CI**	**p-value**	**OR**	**95% CI**	**p-value**	**OR**	**95% CI**	**p-value**
**CA repeats per allele**														
17	1	0	0	0	1.000	n.a.	0.00-inf	0.992	n.a.	0.00-inf	0.993	n.a.	0.00-inf	0.992
18	0	0	0	0	n.a.	n.a.	n.a.	n.a.	n.a.	n.a.	n.a.	n.a.	n.a.	n.a.
19	4	0	1	0	1.000	0.91	0.10-8.58	0.934	48.47	$0.00\text{-}3.48*10^6$	0.496	4.80	0.08-304.56	0.458
20	5	1	1	0	1.000	1.50	0.21-10.64	0.687	0.82	0.10-6.73	0.855	1.33	0.18-10.12	0.781
21	44	14	8	2	0.501	1.60	0.77-3.34	0.209	3.42	1.21-9.70	0.021	1.88	0.81-4.36	0.141
22	9	0	4	0	0.258	0.46	0.13-1.67	0.239	0.33	0.08-1.43	0.140	0.42	0.11-1.67	0.219
23	22	2	4	0	0.850	1.56	0.51-4.77	0.431	1.32	0.39-4.50	0.652	1.66	0.50-5.51	0.405
24	13	1	2	0	0.783	1.74	0.39-7.76	0.468	1.39	0.29-6.68	0.681	1.53	0.33-7.14	0.590
25	28	3	7	3	0.105	0.52	0.25-1.10	0.087	0.38	0.15-0.97	0.044	0.48	0.21-1.12	0.088
26	14	0	5	0	0.344	0.57	0.18-1.82	0.346	0.34	0.09-1.26	0.107	0.39	0.12-1.33	0.132
27	2	0	0	0	1.000	n.a.	0.00-inf	0.993	n.a.	0.00-inf	0.993	n.a.	0.00-inf	0.993
**Large number of CA repeats**														
≥22	46	27	8	10	0.297	0.60	0.30-1.21	0.156	0.26	0.09-0.73	0.010	0.46	0.20-1.05	0.065
≥23	45	23	8	8	0.498	0.75	0.39-1.44	0.385	0.43	0.18-1.05	0.064	0.63	0.30-1.32	0.222
≥24	41	12	8	6	0.253	0.62	0.32-1.20	0.157	0.40	0.18-0.93	0.033	0.51	0.24-1.07	0.074
≥25	40	5	8	5	0.043	0.49	0.24-1.00	0.050	0.28	0.10-0.72	0.009	0.37	0.16-0.86	0.021
≥26	16	0	5	0	0.537	0.67	0.22-2.11	0.497	0.41	0.11-1.52	0.183	0.46	0.14-1.54	0.207
≥27	2	0	0	0	1.000	n.a.	0.00-inf	0.993	n.a.	0.00-inf	0.993	n.a.	0.00-inf	0.993
**Sum of alleles**														
Continuous						0.88	0.74-1.04	0.128	0.73	0.58-0.93	0.010	0.81	0.67-0.99	0.036

Abbreviations. N = total number of participants, OR = odds ratio, CI = confidence interval, ref = reference, PC = principal component. *These data are without 5 patients of whom PCA data were lacking.

Table 10. Logistic regression analysis of *FCGR* genotype on ITI success, in a genetically homogenous European-ancestry population, with or without correction for genetic principal components.

Genotype	Total cohort (N = 113) n (%N)	Success (n=92) n (%)	Failure (n=21) n (%)	Single variant, unadjusted			Number of ref. allele[‡]	Single variant, adjusted PC 1-5[*]			Single variant, adjusted PC 1-3[*]		
				OR	95% CI	p-value		OR	95% CI	p-value	OR	95% CI	p-value
**FCGR2A (p.His166Arg)**													
HH	28 (24.8)	23 (82.1)	5 (18.9)	*ref*	*ref*	*ref*	H	1.28	0.57-2.89	0.547	1.17	0.55-2.50	0.687
HR	58 (51.3)	47 (81.0)	11 (19.0)	0.93	0.29-2.99	0.902							
RR	27 (23.9)	22 (81.5)	5 (18.5)	0.96	0.24-3.77	0.949							
**FCGR2A (p.Gln62Trp)**													
QQ	91 (80.5)	76 (83.5)	15 (16.5)	*ref*	*ref*	*ref*	W	0.33	0.09-1.19	0.091	0.51	0.16-1.63	0.256
QW	22 (19.5)	16 (72.7)	6 (27.3)	0.53	0.18-1.57	0.248							
WW	0 (0.0)	0 (0.0)	0 (0.0)	n.a.	n.a.	n.a.							
**FCGR2B (p.Ile232Thr)**													
II	82 (72.6)	64 (78.0)	18 (22.0)	*ref*	*ref*	*ref*	T	2.84	0.55-14.64	0.213	3.12	0.64-15.21	0.159
IT	31 (27.4)	28 (90.3)	3 (9.7)	2.63	0.72-9.64	0.146							
TT	0 (0.0)	0 (0.0)	0 (0.0)	n.a.	n.a.	n.a.							
**FCGR2C (p.Gln57Ter)[§]**													
STOP	82 (72.6)	66 (80.5)	16 (19.5)	*ref*	*ref*	*ref*	STOP	1.64	0.71-3.79	0.250	1.33	0.63-2.82	0.459
ORF (w/STOP or ncORF)	24 (21.2)	20 (83.3)	4 (16.7)	1.21	0.36-4.04	0.754	ORF	0.77	0.21-2.86	0.693	1.01	0.29-3.55	0.988
ncORF (w/STOP)	6 (5.3)	5 (83.3)	1 (16.7)	1.21	0.13-11.11	0.865	ncORF	0.87	0.25-3.10	0.832	1.15	0.33-4.00	0.822
Missing or missing allele	*1 (0.9)*	*1 (100.0)*	*0 (0.0)*	n.a.	n.a.	n.a.							
**FCGR3A (p.Val176Phe)**													
V[†]	20 (17.7)	15 (75.0)	5 (25.0)	*ref*	*ref*	*ref*	V	0.56	0.25-1.23	0.151	0.72	0.35-1.47	0.367
VF	52 (46.0)	42 (80.8)	10 (19.2)	1.40	0.41-4.77	0.590							
F[†]	41 (36.3)	35 (85.4)	6 (14.6)	1.94	0.51-7.37	0.328							
**FCGR2B promoter haplotypes**													
2B.1	90 (79.6)	74 (82.2)	16 (17.8)	*ref*	*ref*	*ref*	2B.4	0.77	0.19-3.18	0.718	0.88	0.26-2.92	0.835
2B.1/2B.2	1 (0.9)	0 (0.0)	1 (100.0)	n.a.	n.a.	n.a.							
2B.1/2B.4	21 (18.6)	17 (81.0)	4 (19.0)	0.92	0.27-3.10	0.892							
2B.4	1 (0.9)	1 (100.0)	0 (0.0)	n.a.	n.a.	n.a.							
**FCGR2C promoter haplotypes**													

2B.1	88 (77.9)	71 (80.7)	17 (19.3)	*ref*	*ref*	*ref*	2B.2	0.77	0.21-2.86	0.693	1.01	0.29-3.55	0.988
2B.1/2B.2	24 (21.2)	20 (83.3)	4 (16.7)	1.20	0.36-3.96	0.768							
Missing or missing allele	*1 (0.9)*	*1 (100.0)*	*0 (0.0)*	n.a.	n.a.	n.a.							
**FCGR3B haplotypes**													
NA1	14 (12.4)	12 (85.7)	2 (14.3)	*ref*	*ref*	*ref*	NA1	1.13	0.47-2.70	0.786	1.12	0.50-2.51	0.781
NA1/NA2	52 (46.0)	42 (80.8)	10 (19.2)	0.70	0.14-3.64	0.671	NA2	1.10	0.50-2.41	0.810	1.11	0.56-2.23	0.759
NA2	46 (40.7)	37 (80.4)	9 (19.6)	0.69	0.13-3.62	0.656							
Missing or missing allele	*1 (0.9)*	*1 (100.0)*	*0 (0.0)*										

Abbreviations. N = total number of participants, OR = odds ratio, CI = confidence interval, ref = reference, PC = principal component, ORF = open reading frame, ncORF = nonclassic ORF, w/ = with. [†]Allele variation between one or two V or F. [‡]This column shows the reference allele of the logistic regression data, which presents the effect of each number of reference allele on ITI success. [*]These data are without 5 patients of whom PCA data were lacking. [§]Combinations for this SNP were reported as follows: having an ORF allele was dominant, thus people with an ORF allele, with or without a STOP or ncORF allele, were reported under 'ORF (w/STOP or ncORF)'. People with a ncORF allele, with or without a STOP allele were reported under 'ncORF (w/STOP)'. People with only STOP allele(s) were reported under 'STOP'. The combinations for this SNP included: for ORF (ORF/STOP (n=19), ORF/STOP/STOP (n=3), ORF/ncORF (n=1), ORF/ncORF/ncORF (n=1)); for ncORF (ncORF (n=2), ncORF/STOP/STOP (n=1), ncORF/ncORF/STOP (n=2), ncORF/ncORF/STOP/STOP (n=1)); for STOP (STOP (n=12), STOP/STOP (n=64), STOP/STOP/STOP (n=5), STOP/STOP/STOP/STOP (n=1)).

References

1. Rakyan VK, Down TA, Balding DJ, Beck S. Epigenome-Wide Association Studies for common human diseases. Nat Rev Genet. 2011;12(8):529-41.
2. Eckhardt CL, Astermark J, Nagelkerke SQ, Geissler J, Tanck MW, Peters M, et al. The Fc gamma receptor IIa R131H polymorphism is associated with inhibitor development in severe hemophilia A. J Thromb Haemost. 2014;12(8):1294-301.
3. Astermark J, Donfield SM, Gomperts ED, Schwarz J, Menius ED, Pavlova A, et al. The polygenic nature of inhibitors in hemophilia A: results from the Hemophilia Inhibitor Genetics Study (HIGS) Combined Cohort. Blood. 2013;131(8):1446-54.
4. Tomizawa R, Watanabe M, Inoue N, Takemura K, Hidaka Y, Akamizu T, et al. Association of functional GITR gene polymorphisms related to expression of glucocorticoid-induced tumour necrosis factor-receptor (GITR) molecules with prognosis of autoimmune thyroid disease - PMC (nih.gov). Clin Exp Immunol. 2011;165(2):141-47.
5. Pavlova A, Delev D, Laxrois-Desmazes S, Schwaab R, Mende M, Fimmers R, et al. Impact of polymorphisms of the major histocompatibility complex class II, interleukin-10, tumor necrosis factor-alpha and cytotoxic T-lymphocyte antigen-4 genes on inhibitor development in severe hemophilia A. J Thromb Haemost. 2009;7(12):2006-15.
6. Bachelet D, Albert T, Mbogning C, Hässler S, Zhang Y, Schultze-Strasser S, et al. Risk stratification integrating genetic data for factor VIII inhibitor development in patients with severe hemophilia A. PLoS One. 2019;14(6):e0218258.
7. Pinto P, Ghosh K, Shetty S. Immune regulatory gene polymorphisms as predisposing risk factors for the development of factor VIII inhibitors in Indian severe haemophilia A patients. Haemophilia. 2012;18(5):794-7.
8. Lozier J, Rosenberg PS, Goedert JJ, Menashe I. A Case-Control Study Reveals Immunoregulatory Gene Haplotypes That Influence Inhibitor Risk in Severe Hemophilia A. Haemophilia. 2011;17(4):641-49.
9. Wagner M, Jasek M, Karabon L. Immune Checkpoint Molecules—Inherited Variations as Markers for Cancer Risk. Front Immunol. 2020;11:606721.
10. Chen DP, Lin WT, Yu KH. Investigation of the association between the genetic polymorphisms of the co-stimulatory system and systemic lupus erythematosus. Front Immunol. 2022;13:946456.
11. Chen DP, Wen YH, Lin WT, Hsu FP, Yu KH. Exploration of the association between the single-nucleotide polymorphism of co-stimulatory system and rheumatoid arthritis. Front Immunol. 2023;14:1123832.

12. Chu YC, Yu KH, Lin WT, Wang WT, Chen DP. Finding the Common Single-Nucleotide Polymorphisms in Three Autoimmune Diseases and Exploring Their Bio-Function by Using a Reporter Assay. Biomedicines. 2023;11(9):2426.

13. Astermark J, Wang X, Oldenburg J, Berntorp E, lefvert AK, Group MS. Polymorphisms in the CTLA-4 gene and inhibitor development in patients with severe hemophilia A. J Thromb Haemost. 2007;5(2):263-5.

14. Walker EJ, Hirschfield GM, Xu C, Lu Y, Liu X, Lu Y, et al. CTLA4/ICOS gene variants and haplotypes are associated with rheumatoid arthritis and primary biliary cirrhosis in the Canadian population. Arthritis Rheum. 2009;60(4):931-7.

15. Pawlak-Adamska E, Frydecka I, Bolanowski M, Tomkieqics A, Jonkisz A, Karabon L, et al. CD28/CTLA-4/ICOS haplotypes confers susceptibility to Graves' disease and modulates clinical phenotype of disease. Endocrine. 2017;55(1):186-99.

16. Zuccherato LW, Camelo RM, Chaves DG, Rezende SM. Germline variants of the immune checkpoint proteins PD-1, PD-l1 and CTLA-4 and immune tolerance induction outcome in patients with inherited haemophilia A. Haemophilia. 2023;29(5):1366-68.

17. Alqahtani M, Aljuaimlani A, Al-Tamimi J, Alomar S, Mansour L. TIM3 and CTLA4 immune checkpoint polymorphisms are associated with acute myeloid leukemia in Saudi Arabia. Hematology. 2024;29(1):2329024.

18. Fernández-Mestre M, Sánchez K, Balbás O, Gendzekhzadze K, Ogando V, Cebrera M, Layrisse Z. Influence of CTLA-4 gene polymorphism in autoimmune and infectious diseases. Hum Immunol. 2009;70(7):532-5.

19. Wolff ASB, Mitchell AL, Cordell HJ, Short A, Skinningsrud B, Ollier W, et al. CTLA-4 as a genetic determinant in autoimmune Addison's disease. Genes Immun. 2015;16(6):430-6.

20. Liu X, Swen J, Diekstra MHM, Boven E, Castellano D, Gelderblom H, et al. A Genetic Polymorphism in CTLA-4 Is Associated with Overall Survival in Sunitinib-Treated Patients with Clear Cell Metastatic Renal Cell Carcinoma. Clin Cancer Res. 2018;24(10):2350-56.

21. Juran BD, Atkinson EJ, Schlicht EM, Fridley BL, Petersen GM, Lazaridis KN. Interacting alleles of the coinhibitory immunoreceptor genes cytotoxic T-lymphocyte antigen 4 and programmed cell-death 1 influence risk and features of primary biliary cirrhosis. Hepatology. 2008;47(2):563-70.

22. Dias FC, Medina T, Mendes-Junior CT, Dantas RO, Pissetti CW, Rodrigues Junior V, et al. Polymorphic sites at the immunoregulatory CTLA-4 gene are associated with chronic chagas disease and its clinical manifestations. PLoS One. 2013;8(10):e78367.

23. Oertelt S, Kenny TP, Selmi C, Invernizzi P, Podda M, Gershwin ME. SNP analysis of genes implicated in T cell proliferation in primary biliary cirrhosis. Clin Dev Immunol. 2005;12(4):259-63.

24. Qi YY, Zhao XY, Liu XR, Wang YN, Zhai YL, Zhang XX, et al. Lupus susceptibility region containing CTLA4 rs17268364 functionally reduces CTLA4 expression by binding EWSR1 and correlates IFN-α signature. Arthritis Res Ther. 2021;23(1):279.
25. Spena S, Cairo A, Pappalardo E, Gorski MM, Garagiola I, Hassan S, et al. Genetic variants at the chromosomal region 2q21.3 underlying inhibitor development in patients with severe haemophilia A. Haemophilia. 2022;28(2):270-77.
26. Kaartinen T, Lappalainen J, Haimila K, Autero M, Partanen J. Genetic variation in ICOS regulates mRNA levels of ICOS and splicing isoforms of CTLA4. Molecular Immunology. 2007;44(7):1644-51.
27. García-Espinoza JA, Muñoz-Valle JF, García-Chagollán M, Hernández-Bello J, Palafox-Sánchez CA, López-Villalobos EF, et al. ICOS Gene Polymorphisms (IVS1 + 173 T/C and c. 1624 C/T) in Primary Sjögren's Syndrome Patients: Analysis of ICOS Expression. Curr Issues Mol Biol. 2022;44(2):764-76.
28. Wang S, Zhang X, Leng S, Xu Q, Sheng Z, Zhang Y, et al. Immune Checkpoint-Related Gene Polymorphisms Are Associated With Primary Immune Thrombocytopenia. Front Immunol. 2021;11:615941.
29. Kim YO, Kim HJ, Kim SK, Chung JH, Hong SJ. Association of the CD28/CTLA4/ICOS polymorphisms with susceptibility to rheumatoid arthritis. Clin Chem Lab Med. 2010;48(3):345-53.
30. Kim T, Martínez-Bonet M, Wang Q, Hackert N, Sparks JA, Baglaenko Y, et al. Non-coding autoimmune risk variant defines role for ICOS in T peripheral helper cell development. Nat Commun. 2024;15(1):2150.
31. Houssaini H, Bouallegui E, Abidda O, Tahri S, Elloumi N, Hachida H, et al. ICOS gene polymorphisms in systemic lupus erythematosus: A case-control study. Int J Immunogenet. 2023;50(4):194-205.
32. Higuchi T, Oka S, Furukawa H, Nakamura M, Komori A, Abiru S, et al. Association of a single nucleotide polymorphism upstream of ICOS with Japanese autoimmune hepatitis type 1. J Hum Genet. 2017.
33. Castelli L, Comi C, Chiocchetti A, Nicola S, Mesturini R, Giodano M, et al. ICOS gene haplotypes correlate with IL10 secretion and multiple sclerosis evolution. 2007.
34. Haimila K, Turpeinen H, Alakulppi NS, Kyllönen LE, Salmela KT, Partanen J. Association of genetic variation in inducible costimulator gene with outcome of kidney transplantation. Transplantation. 2009;87(3):393-6.
35. Li Y, Hong M, Huang X, Zhong L, Gu Y, Wang D, et al. PD-1 Polymorphisms Are Associated with Susceptibility of Acute Anterior Uveitis in Chinese Population. DNA Cell Biol. 2019;38(2):121-28.

36. Kasamatsu T, Ino R, Takahashi N, Gotoh N, Minato Y, Takizawa M, et al. PDCD1 and CTLA4 polymorphisms affect the susceptibility to, and clinical features of, chronic immune thrombocytopenia. Br J Haematol. 2018;180(5):705-14.

37. Tseng CC, Lin YZ, Lin CH, Li RN, Tsai WC, Ou TT, et al. Genetic and epigenetic alteration of the programmed cell death 1 in rheumatoid arthritis. Eur J Clin Invest. 2019;49(10):e13094.

38. Zhang W, Song Y, Zhang X. Relationship of Programmed Death-1 (PD-1) and Programmed Death Ligand-1 (PD-L1) Polymorphisms with Overall Cancer Susceptibility: An Updated Meta-Analysis of 28 Studies with 60 612 Subjects. Med Sci Monit. 2021;27:e932146.

39. Pang T, Du L, Li F, Liu Y, Ma X, Cao Q, et al. Association of apoptosis genes in PDCD1 but not PDCD1LG2, FAS, and FASLG with pediatric idiopathic uveitis in Han Chinese. Pediatr Res. 2020;87(4):634-38.

40. Wu Y, Li M, Meng G, Ma Y, Ye J, Sun T, Ji C. Immune checkpoint-related gene polymorphisms are associated with acute myeloid leukemia. Cancer Med. 2023;12(18):18588-96.

41. Kawabata M, Inoue N, Watanabe M, Kobayashi A, Hidaka Y, Miyauchi A, Iwatani Y. PD-1 gene polymorphisms and thyroid expression of PD-1 ligands differ between Graves' and Hashimoto's diseases. Autoimmunity. 2021;54(7):450-59.

42. Cruz D, Rodríguez-Romanos R, Gonzáles-Bartulos M, García-Cadenas I, de la Cámera R, Heras I, et al. LAG3 genotype of the donor and clinical outcome after allogeneic transplantation from HLA-identical sibling donors. Front Immunol. 2023;14:1066393.

43. Mahmoudi M, Rezaiemanesh A, Salmaninejad A, Harshini S, Poursani S, Bahrami T, et al. PDCD1 single nucleotide genes polymorphisms confer susceptibility to juvenile-onset systemic lupus erythematosus. Autoimmunity. 2015;48(7):488-93.

44. Liu C, Jiang J, Hu X, Wang F, Shen YM, Yu G, et al. A Promoter Region Polymorphism in PDCD-1 Gene Is Associated with Risk of Rheumatoid Arthritis in the Han Chinese Population of Southeastern China. Int J Genomics. 2014;2014:247637.

45. Taher Tahoori M, Akbar Pourfathollah A, Akhlaghi M, Daneshmandi S, Hosein Nicknam M, Soleimanifar N. Association of programmed cell death-1 (PDCD-1) gene polymorphisms with rheumatoid arthritis in Iranian patients. Clin Exp Rheumatol. 2011;29(5):763-7.

46. Hassani N, Salmaninejad A, Aslani S, Kamali-Sarvestani E, Vessal M. The association between PD-1 gene polymorphisms and susceptibility to multiple sclerosis. Immunol Med. 2023;46(2):69-76.

47. de With M, Hurkmans DP, Oomen-de Hoop E, Lalouti A, Bins A, El Bouazzaoui S, et al. Germline Variation in PDCD1 Is Associated with Overall Survival in Patients with Metastatic Melanoma Treated with Anti-PD-1 Monotherapy. Cancers (Basel). 2021;13(6):1370.

48. El-Khair SMA, Sameer W, Awadallah N, Shaalan D. Programmed cell death 1 gene polymorphism as a possible risk for systemic lupus erythematosus in Egyptian females. Lupus. 2019;28(12):1427-34.

49. Lin SC, Yen JH, Tsai JJ, Tsai WC, ou TT, Liu HW, Chen CJ. Association of a programmed death 1 gene polymorphism with the development of rheumatoid arthritis, but not systemic lupus erythematosus. Arthritis Rheum. 2004;50(3):770-5.

50. Fujisawa R, Haseda F, Tsutsumi C, Hiromine Y, Noso S, Kawabata Y, et al. Low programmed cell death-1 (PD-1) expression in peripheral CD4(+) T cells in Japanese patients with autoimmune type 1 diabetes. Clin Exp Immunol. 2015;180(3):452-7.

51. Fathi F, Sadeghi E, Lofti N, Hafezi H, Ahmadi M, Mozafarpoor S, Motedayyen H. Effects of the programmed cell death 1 (PDCD1) polymorphisms in susceptibility to systemic lupus erythematosus. Int J Immunogenet. 2020;47(1):57-64.

52. Dmitrieva-Zdorova EV, Gabaeva MV, Seregin YA, Bodoev NV, Voronko OE. PDCD1 PD-1.3 polymorphism and allergic bronchial asthma in Russian and Buryat patients. J Asthma. 2017;54(1):46-52.

53. Parakh S, Musafer A, Paessler S, Witkowski T, Li Wai Suen CSN, Tutuka CSA, et al. PDCD1 Polymorphisms May Predict Response to Anti-PD-1 Blockade in Patients With Metastatic Melanoma. Front Immunol. 2021;12:672521.

54. Kroner A, Mehling M, Hemmer B, Rieckmann P, Toyka KV, Mäurer M, Wiendl H. A PD-1 polymorphism is associated with disease progression in multiple sclerosis. Ann Neurol. 2005;58(1):50-7.

55. Anderson AC, Joller N, Kuchroo VK. Lag-3, Tim-3, and TIGIT co-inhibitory receptors with specialized functions in immune regulation. Immunity. 2016;44(5):989-1004.

56. Johnston RJ, Comps-Agrar L, Hackney J, Yu X, Huseni M, Yang Y, et al. The immunoreceptor TIGIT regulates antitumor and antiviral CD8(+) T cell effector function. Cancer Cell. 2014;26(6):923-37.

57. Trivedi P, Jhala G, de George DJ, Chiu C, Selck C, Ge T, et al. TIGIT acts as an immune checkpoint upon inhibition of PD1 signaling in autoimmune diabetes. Front Immunol. 2024;15:1370907.

58. Liu R, Wang X, Chen X, Wang S, Zhang H. TIM-3 rs1036199 polymorphism increases susceptibility to autoimmune diseases: evidence based on 4200 subjects. Biosci Rep. 2018;38(6):BSR20181235.

59. Razi B, Reykandeh SE, Alizadeh S, Amirzargar A, Saghazadeh A, Rezaei N. TIM family gene polymorphism and susceptibility to rheumatoid arthritis: Systematic review and meta-analysis. PLoS One. 2019;14(2):e0211146.
60. Andrzejczak A, Tupikowski K, Tomkiewicz A, Malkiewicz B, Ptaszkowski K, Domin A, et al. The Variations' in Genes Encoding TIM-3 and Its Ligand, Galectin-9, Influence on ccRCC Risk and Prognosis. Int J Mol Sci. 2023;24(3):2042.
61. Zhang R, Li H, Bai L, Duan J. Association between T-Cell Immunoglobulin and Mucin Domain 3 (TIM-3) Genetic Polymorphisms and Susceptibility to Autoimmune Diseases. Immunol Invest. 2019;48(6):563-76.
62. Chae SC, Park YR, Shim SC, Yoon KS, Chung HT. The polymorphisms of Th1 cell surface gene Tim-3 are associated in a Korean population with rheumatoid arthritis. Immunol Lett. 2004;95(1):91-5.
63. Yaghoobi E, Abedian S, Babani O, Izad M. TIM-3 Rs10515746 (A/C) and Rs10053538 (C/A) Gene Polymorphisms and Risk of Multiple Sclerosis. Iran J Public Health. 2016;45(5):644-9.
64. Smith NL, Chen MH, Dehghan A, Strachan DP, Basu S, Soranzo N, et al. Novel associations of multiple genetic loci with plasma levels of factor VII, factor VIII, and von Willebrand factor: The CHARGE (Cohorts for Heart and Aging Research in Genome Epidemiology) Consortium. Circulation. 2010;121(12):1382-92.
65. Swystun LL, Lillicrap D. Genetic regulation of plasma von Willebrand factor levels in health and disease. J Thromb Haemost. 2018;16(12):2375-90.
66. Mondanelli F, Iacono A, Carvalho A, Orabona C, Volpi C, Pallotta MT, et al. Amino acid metabolism as drug target in autoimmune diseases. Autoimmun Rev. 2019;18(4):334-48.
67. Arefayene M, Philips S, Cao D, Mamidipalli D, Desta Z, Flockhart DA, et al. Identification of genetic variants in the human indoleamine 2,3-dioxygenase (IDO1) gene, which have altered enzyme activity. Pharmacogenet Genomics. 2009;19(6):464-76.
68. Garcia-Martínez I, Borràs N, Martorell M, Parra R, Altisent C, Ramírez L, et al. Common Genetic Variants in ABO and CLEC4M Modulate the Pharmacokinetics of Recombinant FVIII in Severe Hemophilia A Patients. Thromb Haemost. 2020;120(10):1395-406.
69. Xin Z, You L, Li J, Na F, Chen M, Song J, et al. Immunogenetic polymorphisms predict therapeutic efficacy and survival outcomes in tumor patients receiving PD-1/PD-L1 blockade. Int Immunopharmacol. 2023;121.
70. Hayashi M, Kouki T, Takasu N, Sunagawa S, Komiya I. Association of an A/C single nucleotide polymorphism in programmed cell death-ligand 1 gene with Graves' disease in Japanese patients. Eur J Endocrinol. 2008;158(6):817-22.

71. Chin IS, Khan A, Olsson-Brown A, Papa S, Middleton G, Palles C. Germline genetic variation and predicting immune checkpoint inhibitor induced toxicity. NPJ Genom Med. 2022;7(1):73.
72. Fabrizio FP, Trombetta D, Rossi A, Sparaneo A, Castellana S, Muscarella LC. Gene code CD274/PD-L1: from molecular basis toward cancer immunotherapy. Ther Adv Med Oncol. 2018;10.
73. Minari R, Bonatti F, Mazzaschi F, Dodi A, Faccinetti F, Gelsomino F, et al. PD-L1 SNPs as biomarkers to define benefit in patients with advanced NSCLC treated with immune checkpoint inhibitors. Tumori. 2022;108(1):47-55.
74. Zou J, Wu D, Li T, Wang X, Liu Y, Tan S. Association of PD-L1 gene rs4143815 C>G polymorphism and human cancer susceptibility: A systematic review and meta-analysis. Pathol Res Pract. 2019;215:229-34.
75. Yeo MK, Choi SY, Seong IO, Suh KS, Kim JM, Kim KH. Association of PD-L1 expression and PD-L1 gene polymorphism with poor prognosis in lung adenocarcinoma and squamous cell carcinoma. Hum Pathol. 2017;68:103-11.
76. Yoshida H, Nomizo T, Ozasa H, Tsuji T, Funazo T, Yasuda Y, et al. PD-L1 polymorphisms predict survival outcomes in advanced non-small-cell lung cancer patients treated with PD-1 blockade. Eur J Cancer. 2021;144:317-25.
77. Mitchell AL, Cordell HJ, Soemedi R, Owen K, skinningsrud B, Wolff AB, et al. Programmed death ligand 1 (PD-L1) gene variants contribute to autoimmune Addison's disease and Graves' disease susceptibility. J Clin Endocrinol Metab. 2009;94(12):5139-45.
78. Polcaro G, Liguori L, Manzo V, Chianese A, Donadio G, Gaputo A, et al. rs822336 binding to C/EBPβ and NFIC modulates induction of PD-L1 expression and predicts anti-PD-1/PD-L1 therapy in advanced NSCLC. Mol Cancer. 2024;23(1).
79. Grenda A, Karawczyk P, Kucharcyk T, Blach J, Reszka K, Chmieleqska I, et al. Impact of copy number variant and single nucleotide polymorphism of the programmed death-ligand 1 gene, programmed death-ligand 1 protein expression and therapy regimens on overall survival in a large group of Caucasian patients with non-small cell lung carcinoma. Oncol Lett. 2021;21(6):449.
80. Ulrich-Merzenich G, Hausen A, Zeitler H, Goldmann G, Oldenburg J, Pavlova A. The role of variant alleles of the mannose-binding lectin in the inhibitor development in severe hemophilia A. Thromb Res. 2019;179:140-46.
81. Freitas Queiroz MA, Menezes Santiago A, Dos Santos Brito WR, Santos Pereira KA, Botelho de Brito W, da Silva Torres MK, et al. Polymorphisms in the MBL2 gene are associated with the plasma levels of MBL and the cytokines IL-6 and TNF-α in severe COVID-19. Front Immunol. 2023;14:1151058.

82. de Alencar JB, Macedo LC, de Barros MF, Rodrigues C, Shinzato AH, Pelissari CB, et al. New associations: INFG and TGFB1 genes and the inhibitor development in severe haemophilia A. Haemophilia. 2015;21(4):e312-6.

83. Swystun LL, Lai JD, Notley C, Georgescu I, Paine AS, Mewburn J, et al. The endothelial cell receptor stabilin-2 regulates VWF-FVIII complex half-life and immunogenicity. J Clin Invest. 2018;128(9):4057-73.

84. Hodeib H, El Amrousy D, Youssef A, Elaskary E, Fouda MH. BAFF rs9514828 gene polymorphism and the risk of the development of inhibitors in children with severe haemophilia A. Haemophilia. 2022;28(3):472-79.

85. Swystun LL, Notley C, Georgescu I, Lai JD, Nesbitt K, James PD, Lillicrap D. The endothelial lectin clearance receptor CLEC4M binds and internalizes factor VIII in a VWF-dependent and independent manner. J Thromb Haemost. 2019;17(4):681-94.

86. Lacroix-Desmazes S, Voorberg J, Lillicrap D, Scott DW, Pratt KP. Tolerating Factor VIII: Recent Progress. Front Immunol. 2020;10:2991.

87. Repessé Y, Peyron I, Dimitrov JD, Dasgupta S, Moshai EF, Costa C, et al. Development of inhibitory antibodies to therapeutic factor VIII in severe hemophilia A is associated with microsatellite polymorphisms in the HMOX1 promoter. Haematologica. 2013;98(10):1650-55.

88. Pacheco-Gonzalez RM, Avila C, Dávila I, García-Sánchez A, Hernández-Hernández L, Benito-Pescador D, et al. Analysis of FOXP3 gene in children with allergy and autoimmune diseases. Allergol Immunopathol. 2016;44(1):32-40.

89. D'Amico F, Fiorito G, Skarmoutsou EG, M., Rossi GA, Tovato C, Bellocchi C, et al. FOXP3, ICOS and ICOSL gene polymorphisms in systemic sclerosis: FOXP3 rs2294020 is associated with disease progression in a female Italian population. Immunobiology. 2018;223(1):112-17.

90. Oldenburg J, Picard JK, Schwaab R, Brackmann HH, Tuddenham EG, Simpson E. HLA genotype of patients with severe haemophilia A due to intron 22 inversion with and without inhibitors of factor VIII. Thromb Haemost. 1997;77(2):238-42.

91. De Barros MF, Herrero JCM, Sell AM, De Melo FC, Braga MA, Pelissari CB, et al. Influence of class I and II HLA alleles on inhibitor development in severe haemophilia A patients from the south of Brazil. Haemophilia. 2012;18(3):e236-40.

92. Pergantou H, Varela I, Moraloglou O, Economou M, Spanou K, Kapsimali Z, et al. Impact of HLA alleles and cytokine polymorphisms on inhibitors development in children with severe haemophilia A. Haemophilia. 2013;19(5):706-10.

93. McGill JR, Simhadri VL, Sauna ZE. HLA Variants and Inhibitor Development in Hemophilia A: A Retrospective Case-Controlled Study Using the ATHNdataset. Front Med. 2021:663396.

94. Hay CR, Ollier W, Pepper L, Cumming A, Keeney S, Goodeve AC, et al. HLA class II profile: a weak determinant of factor VIII inhibitor development in severe haemophilia A. UKHCDO Inhibitor Working Party. Thromb Haemost. 1997;77(2):234-7.
95. Kim JJ, Hong YM, Yun SW, Lee KY, Yoon KL, Han MK, et al. Identification of B-cell-related HSPG2 and CDSN as susceptibility loci for Kawasaki disease. Hum Immunol. 2023;84(10):567-70.
96. Li Q, Zhou ZW, Lu J, Luo H, Wang SN, Peng Y, et al. PD-L1P146R is prognostic and a negative predictor of response to immunotherapy in gastric cancer. Mol Ther. 2022;30(2):621-31.
